# Ginsenoside Re Regulates the Insulin/Insulin-like Growth Factor-1 Signaling Pathway and Mediates Lipid Metabolism to Achieve Anti-Aging Effects in *Caenorhabditis elegans*

**DOI:** 10.3390/molecules30173463

**Published:** 2025-08-22

**Authors:** Qi Chen, Xiaolu Chen, Linzhen Chen, Xue Zhang, Zhuo Yang, Juhui Hao, Zhiqiang Ma

**Affiliations:** 1 Beijing Key Laboratory for Quality Evaluation of Chinese Materia Medica, Beijing University of Chinese Medicine, Beijing 102488, China; chen_qi0823@163.com (Q.C.); chenxiaolu0109@163.com (X.C.); chenlinzhen123@163.com (L.C.); 12138liuli@sina.com (Z.Y.); 2 Traditional Chinese Medicine Processing Technology Inheritance Base of National Administration of Traditional Chinese Medicine, Beijing 102488, China; zhangxuezyhx@163.com; 3 School of Chinese Materia Medica, Beijing University of Chinese Medicine, Beijing 102488, China; ddhl1972@sohu.com

**Keywords:** *Caenorhabditis elegans*, Ginsenoside Re, IIS pathway, lipid metabolism, anti-aging

## Abstract

The increasing demographic aging of society is a great challenge to the healthcare sector and raises the socio-economic burden. Therefore, elucidating the mechanisms of aging and developing safe effective anti-aging products to prolong people’s healthy lifespan are paramount nowadays. *Panax ginseng* has been highly regarded since ancient times for its ability to enhance health and prolong life. However, its main active substances of anti-aging and their mechanisms are not fully understood. In this research, *Caenorhabditis elegans* was used as a model organism to explore and confirm the key active substances from *Panax ginseng* and the mechanisms that exert anti-aging effects. Various ginsenoside compounds were evaluated based on longevity, anti-stress, physiological function, etc. Ginsenoside Re, which has powerful anti-aging activity, was screened. In the follow-up trials, transcriptomics and RT-qPCR techniques were used to investigate the mechanism of Re in exerting its anti-aging properties. Differential genes were enriched in the Insulin/Insulin-like Growth Factor-1 Signaling (IIS) pathway, the neuropeptide signaling pathway, and lipid metabolism. A significant increase in the expression levels of *daf-16*, *sgk-1*, *skn-1*, *hsf-1*, *hsp-16.2*, *sod-3*, *gst-4*, *fil-2*, *lips-11*, *cyp-35A4*, and *aex-2* genes, and a significant decrease in the expression levels of *daf-2*, *age-1*, and *akt-2* genes were verified. These suggest that ginsenoside Re exerts its life-extending influence by regulating lipid metabolism and the IIS pathway.

## 1. Introduction

As living conditions improve, the problem of an aging population due to demographic changes gradually attracts attention. At the same time, exploring aging has become a critical topic for scholars. Aging is a degenerative change in the organism accompanied by the complex process of physiological function decline [1]. In the process of organism aging, the metabolic rate of the organism, adaptability, and resistance decrease leading to an increase in diseases. Aging is one of the factors that contribute to diseases such as cancer, cardiovascular disease, Alzheimer’s disease, and others [2]. Therefore, in the field of anti-aging research, the development of safe and effective medications is a primary goal.

*Caenorhabditis elegans* is a well-established model for investigating aging due to its short life cycle, rapid reproduction, ease of observation [3], fully sequenced genome, and the fact that 40% of its genes are direct homologs of human genes [4]. By measuring various aging markers in the research, we were able to analyze the direct effects of different factors on the nematodes.

*Panax ginseng* is the dried root and rhizome of the *Araliaceae* plant, *Panax ginseng* C. A. Mey. The Chinese classic “*Shennong Bencao Jing*” (*Classic of Herbal Medicine*)—revered as one of the earliest extant pharmacopoeias in China, dating back to approximately the Han Dynasty (c. 200 BCE) and systematically documenting the properties and uses of hundreds of medicinal substances—records that ginseng can lighten the body and prolong life when taken regularly [5]. As a longevity enhancer, it has been known since ancient times for its variety of pharmacological effects [6]. Ginsenosides are the main chemical constituents in *Panax ginseng*. Multiple animal experiments have demonstrated the anti-aging effects of total ginsenosides, including *C. elegans*, mice, and *Drosophila melanogaster* [7,8]. Researchers have isolated numerous individual saponin compounds from total ginseng extracts. In anti-aging studies of ginsenosides, multiple screening approaches have been employed to identify potent bioactive compounds. Wang et al. [9] evaluated the anti-aging activities of total ginsenosides and principal saponin constituents (Rg1, Rg2, Re, Rb1, Rh1, Rh2, Rd, and Rc). Using oxidative stress survival assays and lipofuscin content quantification (where Rg1, Re, and Rb1 showed statistical significance), they demonstrated that Rg1, Re, and Rb1 are the primary active constituents responsible for anti-aging effects. Yu et al. [10] reported significant anti-aging effects of total ginsenosides using *C. elegans.* Among the four most abundant ginsenosides (Rg1, Re, Rg2, and Rd), ginsenoside Rd at 1 μg/mL exhibited senescence-delaying effects comparable to total saponins. Chen et al. [11] screened seven ginsenosides (Rb1, Rb2, Rg1, Rg2, Rg3, Rh1, Rh2, and Rd) for anti-aging activity using Saccharomyces cerevisiae as the model organism. Rg1 and Rg3 demonstrated the most pronounced efficacy in their experimental system. As a ginseng protopanaxatriol saponin, ginsenoside Re exhibits multifaceted bioactivities relevant to aging modulation. In mammalian models, Re attenuates neuroinflammation by promoting PINK1-dependent mitophagy and suppressing NLRP3 inflammasome activation [12]. However, key limitations persist, including restricted diversity of tested saponins, reliance on singular evaluation metrics, inconsistent dosage regimes, and so on.

Therefore, to address the limitations of prior individual ginsenoside anti-aging studies (such as limited compound variety, single evaluation metrics, and inconsistent concentrations), this study employed a standardized screening approach. This work aimed to provide a robust comparative analysis of anti-aging efficacy across diverse ginsenoside structures under unified conditions, and to deliver an integrated and mechanistic understanding of the most promising candidates, thereby informing future development strategies for ginsenoside-based anti-aging interventions.

## 2. Results

### 2.1. Evaluation of Anti-Aging Activities of Different Types of Ginsenosides

#### 2.1.1. Concentration Screening

**Figure 1:**
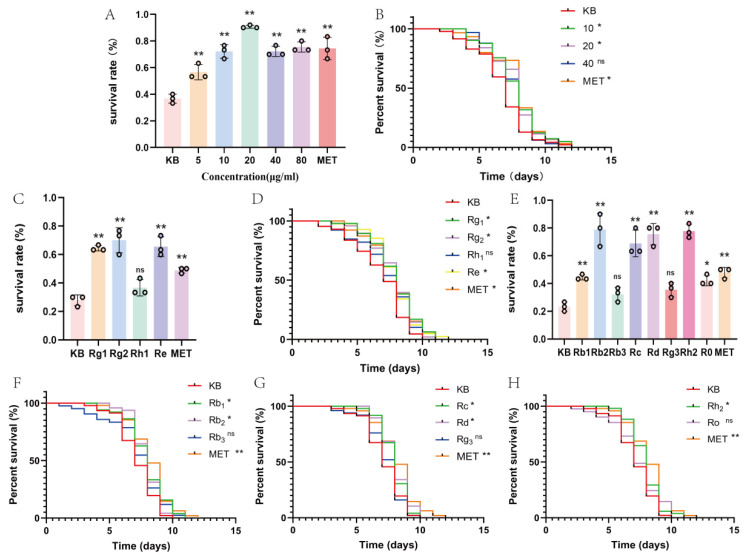
Impact of ginsenosides on heat stress and oxidative stress capacity of *C. elegans*. (**A**) Heat stress capacity of nematodes under the intervention of total ginsenosides. (**B**) Impact of total ginseng saponins on the oxidative stress capacity of *C. elegans*. (**C**) Impact of ginseng of protopanaxatriol saponins on the heat stress capacity of *C. elegans*. (**D**) Impact of ginseng of protopanaxatriol saponins on the oxidative stress capacity of *C. elegans*. (**E**) Impact of ginseng of protopanaxadiol saponins on the heat stress capacity of *C. elegans*. (**F**–**H**) Impact of ginseng of protopanaxadiol saponins on the oxidative stress capacity of *C. elegans*. Ginseng of protopanaxadiol saponins: Rb1, Rb2, Rb3, Rc, Rd, Rg3, and Rh2). Ginseng of protopanaxatriol saponins: Rg1, Rg2, Re, and Rh1. Oleanolic acid-type ginsenoside Ro. Positive control group: MET. * represents *p* < 0.05, indicating significant differences; ** represents *p* < 0.01, indicating highly significant differences.

In this part of the research, we used total ginseng saponins to screen the optimal concentration with Metformin (MET) as the positive control group through the thermal assay (3.5 h) and longevity assay under oxidative stress. According to Figure 1A, total ginseng saponins were able to increase the nematode resistance to heat stress in the range of 5–80 μg/mL, with 20 μg/mL being the most effective. Therefore, a concentration gradient of 10 (Low dose, L), 20 (Medium dose, M), and 40 (High dose, H) μg/mL were selected to determine the administered drug’s concentration in the subsequent trials. In the longevity experiment under oxidative stress conditions (Figure 1B), both MET (80 μg/mL) and 10–20 μg/mL groups significantly prolonged nematode longevity compared to the blank (KB) group (*p* < 0.05). The prolongation rates for each dosing group are shown in Table 1. Therefore, 10 μg/mL was selected as the subsequent administration concentration at which anti-aging activities of various types of saponins were observed.

#### 2.1.2. Longevity Assay Under Oxidative Stress

In this segment of the research, we evaluated the anti-aging activities of ginseng of protopanaxadiol saponins (Rb1, Rb2, Rb3, Rc, Rd, Rg3, and Rh2), ginseng of protopanaxatriol saponins (Rg1, Rg2, Re, and Rh1), and oleanolic acid-type ginsenoside Ro. Stress resistance strongly correlates with longevity extension in *C. elegans* models [13], and stress assays enable rapid candidate identification before committing to lengthy lifespan studies (≥3 weeks). Critically, individual ginsenosides were tested at 10 μg/mL to enable direct comparison with total saponin effects. As later validated, ginsenoside Re at this concentration achieved 16.2% lifespan extension—closely mirroring total saponins’ efficacy (Δ = 1.3%) and confirming dose appropriateness. So, the thermal assay was conducted under 37 °C, while the longevity assay was conducted under oxidative stress, to contrast the anti-aging activities of different types of ginsenosides at a concentration of 10 μg/mL.

In the heat stress experiment of 4 h (Figure 1C,E), Rg1, Rg2, Re, Rb1, Rb2, Rc, Rd, Rh2 and MET were able to increase the nematode resistance to heat stress versus the KB group (*p* < 0.05). In contrast, at the same level, Rh1, Rb3, and Rg3 were not significant, concerning the KB group (*p* > 0.05). In the lifespan experiments under oxidative stress (Figure 1D,F–H), the administration of Rg1, Rg2, Re, Rb1, Rb2, Rc, Rd, Rh2 and MET caused a rightward shift in the nematode survival curves (*p* < 0.05). The mean lifespan and prolongation rates for each dosing group are shown in Table 2 and Table 3, with the better-performing mono saponins being Rg1, Rd, and Re.

#### 2.1.3. Natural Longevity Assay

**Figure 2:**
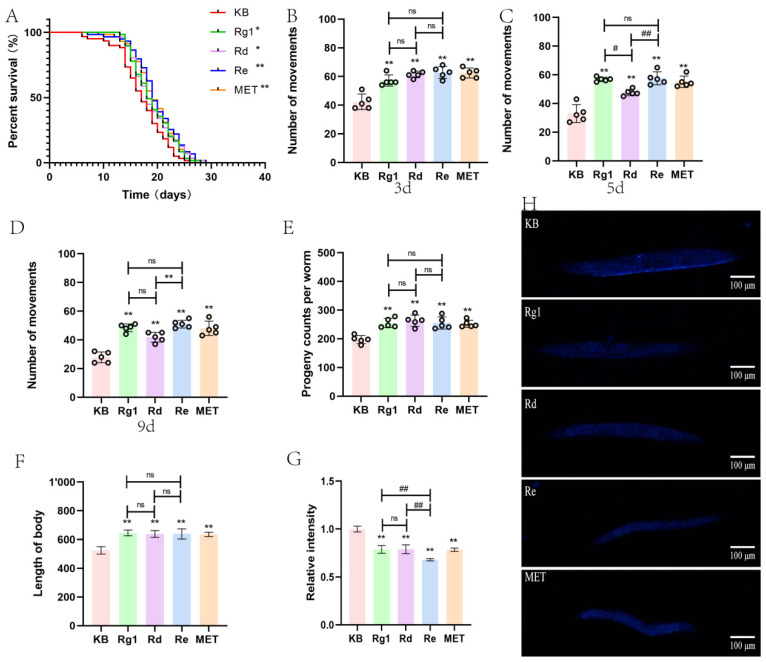
Evaluation of the anti-aging activity of Rg1, Rd, and Re. (**A**) Influence of Rg1, Rd, and Re on *C. elegans* longevity. (**B**–**D**) Influence of Rg1, Rd, and Re on *C. elegans* motility. (**E**) Influence of Rg1, Rd, and Re on *C. elegans* fecundity. (**F**) Influence of Rg1, Rd, and Re on *C. elegans* body length. (**G**,**H**) Influence of Rg1, Rd, and Re on *C. elegans* lipofuscin accumulation. Ginseng of protopanaxadiol saponins: Rd. Ginseng of protopanaxatriol saponins: Rg1, Re. Positive control group: MET. *^, #^ represents *p* < 0.05, indicating significant differences; **^, ##^ represents *p* < 0.01, indicating highly significant differences.

In the previous pilot, we performed a preliminary screening of 12 ginsenosides by heat stress and oxidative stress assay. The results imply that at the level of 10 μg/mL, Rg1, Rd, and Re showed outstanding performance. Therefore, we further compared the anti-aging activities of the three by natural longevity assay. In Figure 2A, Rg1, Rd, Re and MET all shifted the nematode survival curves to the right. Re and MET were highly significant (*p* < 0.01), and Rg1 and Rd were significant (*p* < 0.05) for the prolongation of nematode lifespan different from the KB group. Rg1, Rd, and Re resulted in the prolongation of nematode lifespan by 10.5%, 10.5%, and 15.1%, respectively (Table 4). From the data, it is clear that the extension of nematode lifespan by Re was most pronounced in the treated groups.

#### 2.1.4. Physiological Function Assay

The number of movements in each nematode group for 3, 5, and 9d was measured to see if the administration of the drug would interfere with the nematode’s locomotor ability. As can be seen in Figure 2B–D, all administration groups increased the number of nematode movements (*p* < 0.01). At 5d, the nematodes in the Rd dosing group showed a statistically significant difference from the other dosing groups (*p* < 0.05). At 9d, the worms in the Rd treatment group showed statistical distinct only from the Re group (*p* < 0.01). Nematodes in the Rg1 group did not differ from the other two treated groups although the number of movements decreased (*p* > 0.05). The medicine’s reproductive toxicity was monitored by measuring the number of nematode offspring. In Figure 2E, all administration groups do not inhibit nematode reproduction and, to some extent, increase the number of nematode progenies. As shown in Figure 2F, all dosing groups were able to increase the nematode’s length very significantly, (*p* < 0.01) versus KB, and were able to promote nematode growth and development.

#### 2.1.5. Lipofuscin Accumulation

Lipofuscin, an autofluorescent senescence pigment, is recognized as a quantitative biomarker of cellular aging [14]. Under a fluorescence microscope, lipofuscin in the nematode can be observed to exhibit spontaneous blue fluorescence (Figure 2H). The level of fluorescence of lipofuscin (Figure 2G) in nematodes was reduced in all dosing groups compared to the KB group (*p* < 0.01). Particularly noteworthy was the lower level of lipofuscin in the Re group versus the other two groups (*p* < 0.01). The outcomes showed that all three ginsenosides reduced lipofuscin accumulation in the nematode intestines to delay the degree of nematode senescence, with Re performing the best.

### 2.2. The Anti-Aging Mechanism of Re

Based on anti-aging activity assessments, the ginsenosides demonstrating optimal anti-aging activities were subjected to further mechanistic investigations.

#### 2.2.1. Gene Transcription Analysis

**Figure 3:**
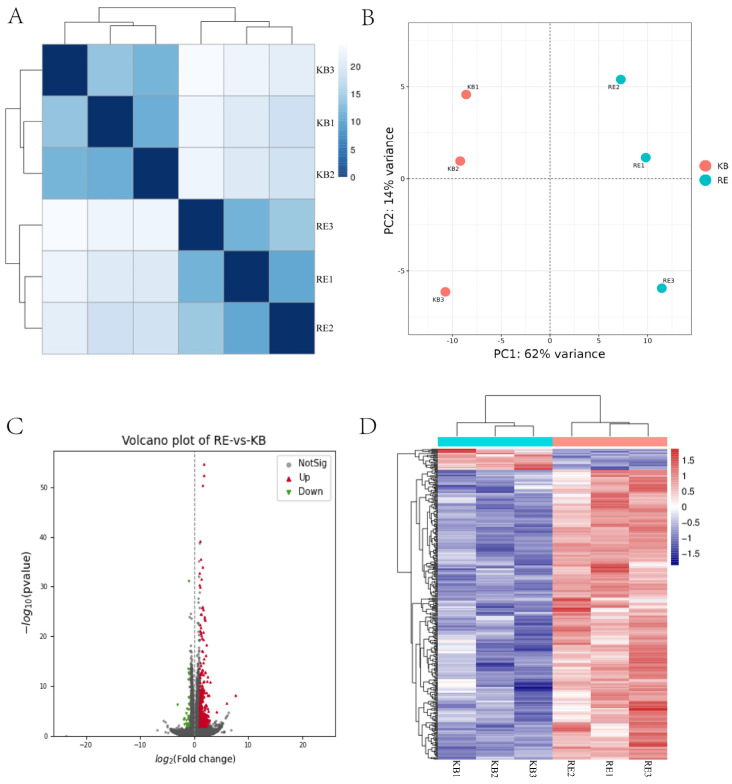
Transcriptology. (**A**) Distance heatmap. (**B**) Inter-sample PCA analysis. (**C**) Volcano plot of differential genes (genes with parameters |log2FC| ≥ 1, *p* < 0.05, and FDR < 0.05 were considered as differentially expressed genes). (**D**) Heatmap of differential genes.

Distance heatmaps and PCA analyses were used to analyze correlations between samples. As indicated by the distance heatmap (Figure 3A), the three biological replicates are clustered together within the group and separated between the groups. This indicates that the sample groups are well-preceded and -differentiated between the groups. PCA analysis (Figure 3B) presented a clustering of three replicates within the group and significant separation of samples between the groups, implying good within-group reproducibility and significant effect after drug treatment. Analysis of the differential genes resulted in a volcano map (Figure 3C), in which 358 genes were up-regulated and 26 genes were down-regulated. At the same time, we also performed clustering (Figure 3D).

**Figure 4:**
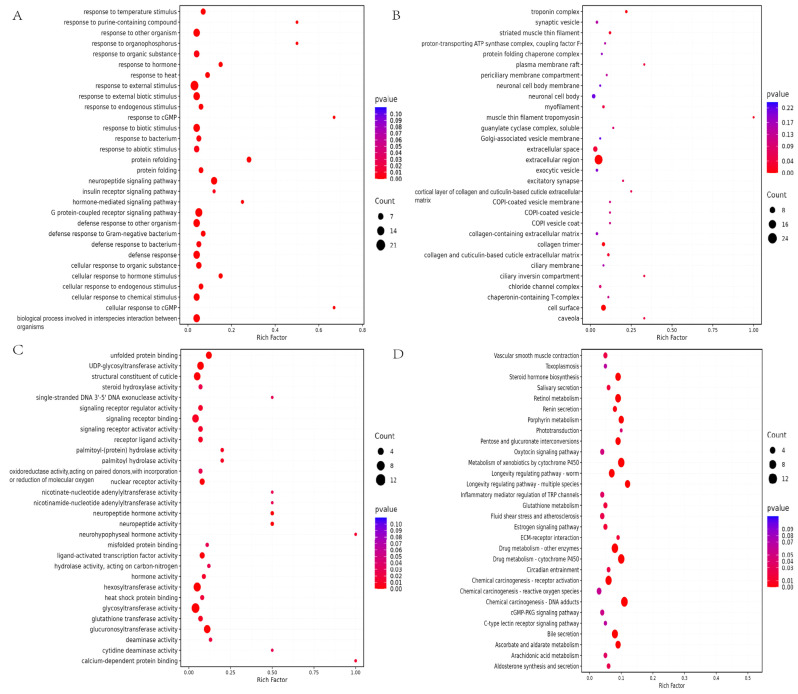
Enrichment analysis. (**A**) Biological Process. (**B**) Cellular Component. (**C**) Molecular Function. (**D**) KEGG analysis. (Threshold: *p* < 0.05.)

GO and KEGG pathways analyses (Figure 4A–D) of the differential genes. In the functional annotation analysis using the GO database, we can see that biological processes such as insulin receptor signaling pathway, neuropeptide signaling pathway, G protein-coupled receptor signaling pathway, and response to external stimuli are enriched in the Biological Process (BP). In the neuropeptide signaling pathway, *aex-2* was upregulated 1.70-fold. The Cellular Component (CC) was enriched for cellular components involved in neural signaling such as synaptic vesicles, neuronal cell body membranes, and excitatory synapses. Relevant molecular functions such as neuropeptide hormone activity, neuropeptide activity, heat shock protein binding, and glutathione transferase activity were enriched in the Molecular Function (MF). The pathways enriched by KEGG PATHWAY analysis were the longevity regulating pathway—worm, longevity regulating pathway—multiple species, glutathione metabolism, and arachidonic acid metabolism, among other related signaling pathways. In the longevity regulating pathway—worm, *hsp-16.2* showed a 2.51-fold upregulation, and *fil2* (encoding a lipase) was upregulated 2.24-fold.

#### 2.2.2. Re Exerts Anti-Aging Effects Through the Insulin/Insulin-like Growth Factor-1 Signaling (IIS) Pathway

**Figure 5:**
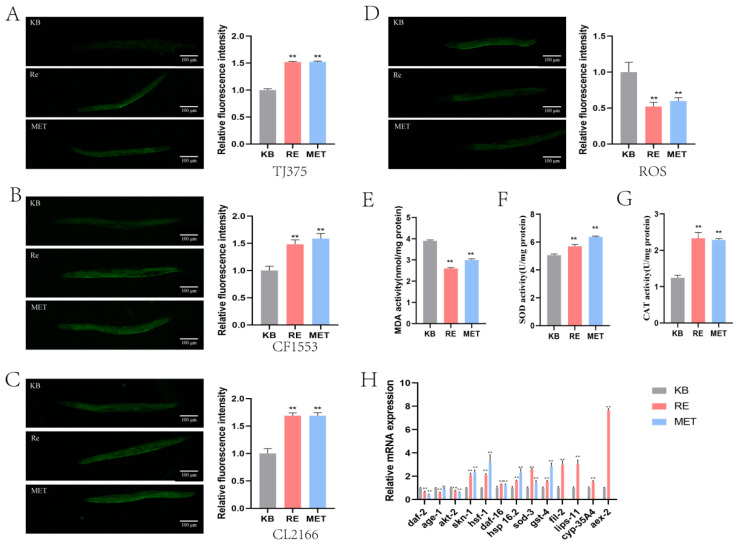
Re exerts anti-aging effects through the IIS pathway and lipid metabolism. (**A**) TJ375 *C. elegans* HSP-16.2: GFP fluorescence. (**B**) CF1553 *C. elegans* SOD-3::GFP fluorescence. (**C**) CL2166 *C. elegans* GTS-4::GFP fluorescence. (**D**) ROS levels in *C. elegans* after Re intervention. (**E**) MDA levels in *C. elegans* after Re intervention. (**F**) SOD levels in *C. elegans* after Re intervention. (**G**) CAT levels in *C. elegans* after Re intervention. (**H**) Influence of Re on the expression of related genes in nematodes in vivo. Ginseng of protopanaxatriol saponins: Re. Positive control group: MET. ** represents *p* < 0.01, indicating highly significant differences.

In nematodes, following the Re intervention, we detected that superoxide dismutase (SOD) and catalase (CAT) viability (Figure 5F,G) were higher, versus the KB group (*p* < 0.01). In contrast, reactive oxygen species (ROS) and malondialdehyde (MDA) levels (Figure 5D,E) in nematodes were decreased after Re intervention (*p* < 0.01). This suggests that Re enhances the antioxidant capacity of nematodes and attenuates oxidative damage, thereby affecting the aging process. The *Hsp-16.2* on the IIS pathway is able to encode heat shock proteins involved in the heat stress response. The *Sod-3*, located in mitochondria, is a superoxide dismutase regulated by DAF-16 which plays an important role in ROS detoxification [15]. When the organism is under oxidative stress due to ROS overproduction, the transcription of antioxidant genes such as *sod-3* and *gst-4* is enhanced, due to the release of DAF-16 and SKN-1 from the cytoplasm to the nucleus [16]. During fluorescence measurements of the relevant genes in the transgenic nematode (Figure 5A–C and Table 5), we found elevated levels of HSP-16.2, SOD-3, and GST-4 (*p* < 0.01).

Through RT-qPCR, we examined the expression levels of key genes in the IIS pathway. The results of the investigation showed a significant increase in the expression levels of the lifespan-related genes *daf-16*, *skn-1*, *hsf-1*, *hsp-16.2*, *sod-3*, and *gst-4*, and a significant decrease in the expression levels of the *daf-2*, *age-1*, and *akt-2* genes, following the intervention of Re (Figure 5H and Table 6). This demonstrates once again that Re exerts its age-delaying effects through the IIS pathway.

#### 2.2.3. Re Exerts Longevity by Regulating Lipid Metabolism

In addition to the classical signaling pathways associated with longevity, we were surprised to find differential gene enrichment in the neuropeptide signaling pathway, as well as lipid metabolism bioprocesses. Expression levels of neuropeptide family-related genes (*nlp-15, nlp-43, nlp-3, nlp-55, nlp-13, nlp-59, nlp-82, nlp-50, flp-17*, and *flp-25*) were detected upregulated by transcriptomics. Meanwhile, we utilized RT-qPCR to validate the lipid metabolism-related genes (*fil-2*, *lips-11,* and *cyp-35A4*) and *aex-2* genes enriched in transcriptomics. And the up-regulation of *fil-2*, *lips-11,* and *cyp-35A4*, and *aex-2* gene expression levels were also verified again, by us, in the experiment (Figure 5H and Table 6).

## 3. Discussion

Our systematic evaluation of 12 structurally diverse ginsenosides reveal critical insights obscured by prior fragmented approaches. This broad-spectrum screen identified multiple high-potency saponins. Among the ginseng of protopanaxatriol saponins, Re (featuring glucose at C_6_ and a glucose–rhamnose pair at C_20_) emerged as the supreme longevity enhancer—its unique sugar arrangement likely enabling superior target engagement. Meanwhile, Rd showed the strongest anti-aging effects in the ginseng of protopanaxadiol saponins, while Rg3 demonstrated specialized heat resistance uncorrelated with lifespan. These demonstrate that comprehensive *Panax ginseng* compound screening is a gateway to discover potent anti-aging agents.

The highly conserved IIS pathway is a key pathway in the control of organismal development and senescence. From simple invertebrates to mammals, the IIS pathway regulates lifespan in many animals, e.g., nematodes, Drosophila, mice, and humans. When the IIS pathway is activated under favorable conditions, agonist insulin-like peptides (ILPs) bind to the receptor DAF-2 to activate AGE-1 [17]. At this point, elevated levels of phosphatidylinositol 3,4,5-trisphosphate (PIP3), in turn, activate the downstream kinase cascade reaction. These kinases, consisting of PDK-1, AKT-1, AKT-2, and SGK-1, promote the phosphorylation of the DAF-16/FOXO transcription factor and prevent DAF-16 from translocating and inactivating it [18]. When in unfavorable conditions, the IIS pathway is inhibited. After dephosphorylation, DAF-16/FOXO transcription factors translocate from the cytoplasm to the nucleus to perform transcriptional regulatory functions and induce long-lived gene expression [19]. This process increases the resistance of nematodes to various stresses, and prolongs lifespan.

The three most important transcription factors downstream of the IIS pathway include DAF-16/FOXO, HSF-1, and SKN-1/Nrf2. When the body is thermally stimulated, a heat shock response occurs, which in turn activates HSF-1 to induce transcription of genes encoding heat shock proteins (HSPs) and molecular chaperones [20,21]. DAF-16 also plays a role in the heat shock response, in which HSP-1, together with DAF-16, activates the expression of the small heat shock protein gene [22]. A homolog of the mammalian Nr2f protein, SKN-1 is a regulator capable of participating in antioxidant and oxidative stress defenses [23]. When subjected to oxidative stress, or when the activity of the IIS pathway is reduced, phosphorylated SKN-1 undergoes nuclear translocation to induce the expression of relevant detoxification genes (*gat-1*, *sod-1*, and *gcs-1*), thereby counteracting oxidative stress [24]. Re improved nematode stress resistance, reduced lipofuscin accumulation, lowered ROS and MDA content in the body, and increased the activity of antioxidant enzymes SOD and CAT. At the same time, Re was able to up-regulate the expression levels of *daf-16*, *skn-1*, *hsf-1*, *hsp-16.2*, *sod-3*, and *gst-4* genes, down-regulating the expression levels of the *daf-2*, *age-1*, and *akt-2* genes in nematodes. This suggests that Re works through the IIS pathway to enhance oxidative defense and stress resistance in nematodes, thereby exerting a delayed aging effect.

Changes in lipid metabolism are associated with aging-related diseases [25]. The level of lipid metabolism is critical for physiological or pathological changes in organisms. Neuropeptides are a special class of informational substances, broadly defined as endogenous active substances present in neural tissue and involved in the functional actions of the nervous system. Several studies have shown that neuropeptides and lipid metabolism are closely related to *C. elegans* lifespan regulation. Savini et.al report a fat-to-neuron lipid signaling pathway (NLP-11 neuropeptide) induced by lysosomal metabolism and its longevity-promoting role in *C. elegans* [26]. Another research study identified a neuropeptide FLP-7 with a neuronal secretion that can be detected by the G protein-coupled receptor NPR-22, which triggers intestinal fat loss to regulate lipid metabolism [27]. AEX-2 is a G protein-coupled receptor, which may be an important receptor for the action of related neuropeptides. Related surveys have shown that CYP-35A4 acts on the arachidonic acid metabolic pathway and can mediate the conversion of arachidonic acid to 15 (S)-hPETE. Similarly, lips-11, a member of the lipase family lipl/lips, is involved in a variety of lipid metabolic processes [28]. Lipase fil-2 expression is also regulated by the ER protein IRE-1 and the ER hapten HSP-4, which can promote lipid metabolism [29]. In the mechanism of lipid metabolism, which regulates longevity, we enriched for differential genes of the neuropeptide family (NLPs and FLPs) and detected up-regulation of the expression level of the lipid metabolism-related gene *fil-2*, *lips-11*, and *cyp-35A4*. The important G protein-coupled receptor AEX-2 was also identified. Although exactly which neuropeptide interacts with lipid metabolism via the receptor AEX-2 is unclear, this is a good research direction at this point, regarding synergistic metabolic and neurological anti-aging. All in all, our data demonstrate that Re extends lifespan by targeting the IIS pathway, notably through downregulation of *daf-2* and upregulation of *daf-16*, which enhances oxidative stress resistance. Concurrently, Re upregulates the lipase gene *fil-2*—a key effector in lipid metabolism linked to ER stress responses. While the exact neuropeptide-AEX-2 interaction requires further study, our findings position *daf-2*, *daf-16*, and *fil-2* as central nodes in the synergistic regulation of metabolism and longevity.

Although locomotion and lipofuscin assays were employed using independent synchronous cohorts (Section 4.4) to minimize confounders, we acknowledge that extended lifespan may create a permissive context for improved physiological metrics. For example, reduced lipofuscin accumulation could reflect delayed organismal senescence, rather than direct Re-mediated lipolysis. Future studies using short-lived mutants will decouple lifespan effects from specific anti-aging mechanisms. Although this study establishes that ginsenoside Re extends lifespan through coordinated regulation of the IIS pathway and lipid metabolism, we recognize that the precise crosstalk mechanisms remain incompletely resolved. The absence of genetic validation using mutant strains (e.g., *daf-16* null, *aex-2* knockdown) limits definitive causal attribution. Future work will employ targeted genetic interventions and single-cell transcriptomics to decipher how neuropeptide signaling receptors (e.g., AEX-2) modulate the expression and function of lipid metabolism genes (*fil-2*, *lips-11*, and *cyp-35A4*) identified in our transcriptomic analysis, and explore clinical translatability in mammalian models.

## 4. Materials and Methods

### 4.1. Materials

#### 4.1.1. Chemicals and Regents

Total saponin of *Panax ginseng* (82.56%) (batch number D12IS234679) was obtained from Shanghai Yuanye Bio-Technology Co., Ltd. (Shanghai, China). Ginsenoside Rb1 (98.88%), Rb2 (98.50%), Rb3 (99.86%), Rc (99.37%), Rd (98.54%), Re (99.08%), Rg1 (98.59%), Rg2 (99.57%), Rg3 (99.83%), Rh1 (98.33%), Rh2 (99.59%), and Ro (99.20%) (batch number 230614, 231018, 230812, 230618, 230712, 230723, 230821, 230614, 230921, 230620, 230715, and 230809) were obtained from Plant Standard Pure Biotechnology Co., Ltd (Chengdu, China). All relevant information about the tested ginsenosides can be found in Supplementary Material S1. Assay kits for BCA (batch number 20240620), MDA (batch number 20240621), SOD (batch number 20240613), and CAT (batch number 20240621) were purchased from Nanjing JianCheng Bioengineering Institute (Nanjing, China). Trizol, All-in-One First-Strand Synthesis MasterMix, 2× Realab Green PCR Fast mixture (batch number 100002041, 0202120731, and 0202012341) were supplied by LABLEAD Co. (Beijing, China). The Reactive Oxygen Species Assay Kit (batch number BL714A) was purchased from Ranjeko Technology Co. (Beijing, China). Methylviologen (batch number C16470923), 5-Fluoro-2′ -Deoxyuridine (batch number C12760674), and 1-Phenoxy-2-propanol (batch number M15218018) were acquired from Macklin Biochemical Technology Co. (Shanghai, China). SMZ745 body microscope and TS-2 fluorescence inverted microscope were purchased from Nikon (Tokyo, Japan). All the other chemicals and solvents used in this study were purchased from local reagent providers, and were analytical or chromatographical grade.

#### 4.1.2. Nematode Strain

All *C. elegans* strains used in this experiment were from the *Caenorhabditis* Genetics Center (University of Minnesota, Minnesota, MN, USA), with names and genotypes listed in Table 7. All strains of nematodes were cultured at 22 °C and fed live *E. coli* OP50.

### 4.2. Nematode Culture Methods

In accordance with Reference [30], the experimental conditions were adjusted. Nematode growth medium (NGM) solid medium was used to culture nematodes in this research. After spreading the drug-OP50 mixture, plates were dried for 15 min at 22 °C until achieving a uniform matte surface. During oviposition, nematodes were collected into EP tubes using M9 buffer. The collected nematodes obtained were centrifuged to remove the supernatant. The EP tubes were vortexed with lysate (H_2_O: NaClO: NaOH: 8:1:1) for four minutes and then washed three times with M9 buffer to clean the eggs. Eggs were transferred to fresh NGM medium and incubated at 22 °C for 48 h to reach the L4 stage for subsequent experiments.

### 4.3. Preparation for Administration

Test compounds were accurately weighed and dissolved in DMSO to prepare stock solutions (500 μg/mL). These stock solutions were subsequently serially diluted in *E. coli* OP50 bacterial suspension to achieve the desired concentrations. Each dilution was evenly spread onto fresh NGM plates and allowed to dry prior to nematode culture. The nematodes in the KB group were fed with *E. coli* OP50 without drugs, and the positive control group MET was administered 80 μg/mL, a concentration optimized through preliminary dose–response experiments in our laboratory. The nematodes in the drug group were fed with *E. coli* OP50 with different drugs, and the content of DMSO in the bacterial solution was 0.1% in each group. Surface administration may under-represent bioavailable doses due to agar diffusion and bacterial metabolism.

### 4.4. Anti-Aging Activity Assessment

#### 4.4.1. Thermal Assay

To prevent nematode egg laying from affecting subsequent counts, NGM plates containing 300 µM pentafluorouracil were used in this assay. L4-stage nematodes were transferred to NGM plates coated with *E. coli* OP50 bacterial solution (with or without drug). L4-stage nematodes were transferred to new NGM plates with at least 30 worms per plate. Total saponin of *Panax ginseng* was used as experimental drugs, and the concentration gradients were set as 5, 10, 20, 40 and 80 μg/mL. MET (80 μg/mL) was used as the trial drug in all positive groups in this research. Three days after administration, the nematodes were picked out and placed on a new plate. The Petri dishes were placed in a constant-temperature incubator at 37 °C [31]. Nematode survival was calculated after a few hours.

#### 4.4.2. Longevity Assay Under Oxidative Stress

The experimental conditions were adjusted in accordance with Reference [32]. A longevity assay under oxidative stress conditions can be used for rapid chemical screening. Three days after the L4-stage nematodes were administered the drug, they were transferred to NGM plates containing 10 mM paraquat and incubated at 22 °C, with one plate per group with a minimum of 30 worms per plate. Survival/death was counted every 24 h until all nematodes died.

#### 4.4.3. Natural Longevity Assay

The longevity assay in nematodes is the preferred indicator for evaluating the ability of the medicines to delay nematode senescence, under identical treatment conditions to Section 4.4.1. One plate of 60 worms per group was placed in the incubator, which was recorded as Day 0 of the longevity trial [33]. Nematode death, loss, and survival were recorded every 24 h. Surviving nematodes were transferred to freshly prepared NGM plates containing the same drug–bacterial mixture at identical concentrations to ensure continuous compound exposure throughout the assay. This transfer process was repeated daily, until all nematodes died.

#### 4.4.4. Physiological Function Assay

Reproductive Capacity Assay

Reproductive capacity was determined according to Han et al. [34]. Nematodes cultured to the L4 stage were transferred to NGM plates coated with *E. coli* OP50 bacterial solution, containing different drugs. Five worms in each group were placed at 22 °C. Worms were transferred to new NGM plates every 24 h, until the nematodes no longer laid eggs. The NGM plates containing eggs were incubated for 48 h. The number of zygotes was counted and the total number of eggs laid was summarized.

Body Length Assay

In accordance with Reference [35], the experimental conditions were adjusted. Three days after the L4-stage worms were dosed, the nematodes were transferred to EP tubes by rinsing with M9. There was one plate per group, with a minimum of 30 worms per plate. The nematodes were anesthetized after washing them three times using an M9 solution. Nematode body length was measured under a microscope.

Exercise of Capacity-Testing Assay

The experimental conditions were adjusted in accordance with Reference [36]. Nematode motility was observed on 3, 5, and 9d after administration. Worms were picked onto fresh NGM medium and M9 solution was added, dropwise. After acclimatization for 20 s, the number of nematode body bends in 30 s was counted, taking the nematode body completing one sinusoidal S-curve as one body bend.

#### 4.4.5. Measurement of Lipofuscin Accumulation

Since the content of lipofuscin in the intestine of nematodes increases with age, the level of lipofuscin accumulation can reflect nematode senescence [37]. After 5 days of exposure to the medications, *C. elegans* were collected and washed with the M9 solution. Anesthetized nematodes were photographed with a fluorescence microscope. The content of lipofuscin was measured using Image J 1.8.0 software (National Institutes of Health, Bethesda, MD, USA).

### 4.5. Anti-Aging Mechanisms of Re

#### 4.5.1. Transcriptomic Analysis of *C. elegans*

Total mRNA was extracted using Trizol from samples (~10,000), which were *C. elegans* exposed to the drug Re for 5d. RNA samples were tested for concentration and purity using Nanodrop 8000 (Thermo Fisher Scientific, Wilmington, DE, USA), agarose gel electrophoresis, and Agilent 4200 (Agilent Technologies, Palo Alto, CA, USA). Eukaryotic mRNA was enriched with oligo (dT) using magnetic beads, after testing the RNA samples. After the mRNA was fragmented by adding a fragmentation buffer, a single strand of cDNA was synthesized using mRNA as a template with six-base random hexamers. Further, after synthesis of two-stranded cDNA using buffers, dNTPs, and DNA polymerase I and RNase H, double-stranded cDNA was purified using AMPure XP beads. The purified double-stranded cDNA was subjected to end repair, the addition of A-tail, connection of sequencing junctions, and then fragment-size selection, using AMPure XP beads. The final library was obtained by PCR amplification of the products obtained from the above operations and purification using AMPure XP beads. PE150 was sequenced on the Illumina HiSeq 2500 high-throughput sequencing platform after library quality control. After quality control of the library, PE150 sequencing was performed using the Illumina HiSeq 2500 high-throughput sequencing platform. Sequencing was performed by Guangdong Magigene Technology Co., Ltd. (Guangdong, China).

After obtaining the sequencing data, the quality control software fast (available online: https://github.com/OpenGene/fastp (accessed on 4 August 2025)) was used to filter the low-quality sequencing data. Next, Bowtie 2 (https://github.com/BenLangmead/bowtie2 (accessed on 4 August 2025)) software aligned the QC sequencing data with ribosomal sequences from the NCBI RefSeq (https://www.ncbi.nlm.nih.gov/refseq/ (accessed on 4 August 2025)) and Rfam (https://rfam.org/ (accessed on 4 August 2025)) databases. The filtered data was obtained by using samtools (http://www.htslib.org/ (accessed on 4 August 2025)) to statistically compare the results, removing the sequences on the comparison. Transcript assembly work was performed by comparing reference genomic information via StringTie (http://ccb.jhu.edu/software/stringtie (accessed on 4 August 2025)). The Salmon software (https://github.com/COMBINE-lab/salmon (accessed on 4 August 2025)) and the R package (1.36.1) tximport (http://www.bioconductor.org/packages/release/bioc/html/tximport.html (accessed on 4 August 2025)) were utilized to obtain the read counts of the genes. Based on the expression matrix after standardization, we analyzed the correlation between the samples. After obtaining read counts of all samples, we used the differential analysis software DESeq2 (http://www.bioconductor.org/packages/release/bioc/html/DESeq2.html (accessed on 4 August 2025)) to perform differential expression analysis of genes, as well as functional over-representation analysis. Genes with parameters |log2FC| ≥ 1, *p* < 0.05, and FDR < 0.05 were considered as differentially expressed genes. In this analysis, we used GO (Gene Ontology; www.geneontology.org/ (accessed on 4 August 2025)) and KEGG (Kyoto Encyclopedia of Genes and Genomes; www.genome.jp/kegg/ (accessed on 4 August 2025)) databases for functional enrichment analysis.

#### 4.5.2. Fluorescence Quantification of Transgenic *C. elegans*

The transgenic nematodes used in this research were TJ375, CF1553, and CL2166. The nematodes were synchronized, grown to the L4 stage, and transferred to NGM plates containing the drug. After 5 days the washed and anaesthetized nematodes were photographed under a fluorescence microscope. The photographed images were manipulated to count the fluorescence intensity, using Image J.

#### 4.5.3. ROS Fluorescence Intensity Measurement Assay

ROS are reactive chemicals containing oxygen free radicals that are involved in cell growth, proliferation, development, differentiation, senescence, apoptosis, and many physio-pathological processes [38]. In accordance with Reference [39], the experimental conditions were modified. The nematode precipitate left after rinsing with M9 buffer was added to DCFH-DA solution at a concentration of 100 μM stained at room temperature and protected from light. After 2 h, the nematodes were washed with M9 buffer. The ROS fluorescence of the anesthetized nematodes was photographed by fluorescence microscope, and the fluorescence intensity was counted by Image J.

#### 4.5.4. Kit Assay

Oxidative damage is caused by excessive accumulation of ROS, which is controlled by SOD and CAT. MDA is also a common indicator of lipid peroxidation levels [40]. After 5 days of L4-stage nematode administration, they were collected and rinsed with M9 buffer, to remove residual *E. coli* OP50. Nematode samples were stored at −80 °C for use. The antioxidant enzyme activity and MDA content in nematodes were measured under the guidance of the kit instructions.

#### 4.5.5. RT-qPCR Assay

Nematodes at the L4 stage were collected by placing them in drug-containing or drug-free medium for 5 days. RNA was extracted using the Trizol method in this experiment, and subsequent operations were performed according to the kit instructions. The primers were listed in Table 8.

#### 4.5.6. Statistical Analyses

All experiments were performed in three biological replicates. Survival analyses were performed using the Kaplan–Meier method by GraphPad Prism 8.0 software (San Diego, CA, USA) and the statistical analyses were conducted using SPSS 20.0 (Inc., Chicago, IL, USA). Data were analyzed and processed using GraphPad Prism 8.0. Significant comparisons were made using a one-way ANOVA and T-test. * and ** indicate significant differences compared to the KB group.

## 5. Conclusions

In this research, we systematically evaluated the in vivo anti-aging potential of 12 representative ginsenoside compounds belonging to three distinct structural classes—ginseng of protopanaxadiol saponins (Rb1, Rb2, Rb3, Rc, Rd, Rg3, and Rh2), ginseng of protopanaxatriol saponins (Rg1, Rg2, Re, and Rh1), and the oleanolic acid-type ginsenoside Ro—under standardized conditions. Specifically, all compounds were tested at the same concentration in *C. elegans* models using parallel assays for survival under heat stress and longevity under oxidative stress. The primary goal of this initial phase was to identify the most potent anti-aging candidates within each structural class. Subsequently, the top-performing ginsenosides identified from this screen underwent a comprehensive mechanistic evaluation. This secondary phase utilized a multi-faceted approach, incorporating diverse physiological and molecular indicators (e.g., stress resistance assays, physiological function assays, antioxidant enzyme activity) to elucidate their anti-aging activities and underlying molecular mechanisms. Among these candidates, the core anti-aging substances in ginsenosides are Rg1, Rd, and Re. Further comparison revealed that Re emerged as the optimal longevity enhancer with the most potent lifespan extension and stress resistance. Our mechanistic dissection revealed that Re achieves life-extending effects by modulating the IIS pathway and mediating lipid metabolism. This work provides precise directions for developing evidence-based ginseng anti-aging products. Looking forward, validating Re’s efficacy in mammalian models and developing targeted delivery systems will accelerate clinical translation.

## Figures and Tables

**Table 1:** Effects of total ginseng saponins on *C. elegans* at longevity assay under oxidative stress.

Sample	Average Lifespan/d	Max Lifespan/d	Life Extension Rate/%	*p*-Value vs. KB
KB	6.7 ± 0.3	12	—	—
L	7.7 ± 0.3	12	14.9	0.032 *
M	7.6 ± 0.3	12	13.4	0.028 *
H	7.5 ± 0.3	11	11.9	0.127
MET	7.7 ± 0.4	12	14.9	0.020 *

Data are expressed as the mean ± SEM (*n* = 3), * *p* < 0.05.

**Table 2:** Effect of ginseng of protopanaxadiol saponins on *C. elegans* at longevity assay under oxidative stress.

Sample	Average Lifespan/d	Max Lifespan/d	Life Extension Rate/%	*p*-Value vs. KB
KB	7.2 ± 0.2	10	—	—
Rb1	7.9 ± 0.2	11	9.7	0.013 *
Rb2	7.9 ± 0.2	10	9.7	0.032 *
Rb3	7.3 ± 0.3	11	1.4	ns
Rc	7.9 ± 0.2	10	9.7	0.028 *
Rd	8.0 ± 0.2	10	11.1	0.014 *
Rg3	7.3 ± 0.2	10	1.4	ns
Rh2	7.9 ± 0.2	11	9.7	0.030 *
Ro	7.3 ± 0.3	10	1.4	ns
MET	8.2 ± 0.2	12	13.9	0.001 **

Data are expressed as the mean ± SEM (*n* = 3), * *p* < 0.05, ** *p* < 0.01.

**Table 3:** Effect of ginseng of protopanaxatriol saponins on *C. elegans* at longevity assay under oxidative stress.

Sample	Average Lifespan/d	Max Lifespan/d	Life Extension Rate/%	*p*-Value vs. KB
KB	6.8 ± 0.3	10	—	—
Rg1	7.9 ± 0.3	11	16.2	0.012 *
Rg2	7.8 ± 0.3	11	14.7	0.015 *
Re	7.9 ± 0.3	12	16.2	0.030 *
Rh1	7.3 ± 0.3	10	7.4	ns
MET	7.8 ± 0.3	11	14.7	0.024 *

Data are expressed as the mean ± SEM (*n* = 3), * *p* < 0.05.

**Table 4:** Effect of Rg1, Rd, and Re on *C. elegans* at natural longevity assay.

Sample	Average Lifespan/d	Max Lifespan/d	Life Extension Rate/%	*p*-Value vs. KB
KB	17.2 ± 0.6	26	—	—
Rg1	19.0 ± 0.5	28	10.5	0.039 *
Rd	19.0 ± 0.5	29	10.5	0.045 *
Re	19.8 ± 0.6	29	15.1	0.001 **
MET	19.5 ± 0.5	27	13.4	0.007 **

Data are expressed as the mean ± SEM (*n* = 3), * *p* < 0.05, ** *p* < 0.01.

**Table 5:** Relative fluorescence intensity of different types of nematodes.

Nematode Strain	KB	RE	MET
TJ375	1.00 ± 0.02	1.52 ± 0.01 **	1.52 ± 0.01 **
CF1553	1.00 ± 0.08	1.48 ± 0.09 **	1.59 ± 0.09 **
CL2166	1.00 ± 0.09	1.69 ± 0.05 **	1.69 ± 0.06 **
N2 (ROS)	1.00 ± 0.14	0.52 ± 0.06 **	0.60 ± 0.05 **

Data are expressed as the mean ± SEM (*n* = 3), ** *p* < 0.01.

**Table 6:** Expression levels of genes.

Gene	KB	RE	MET
*daf-2*	1.00 ± 0.04	0.65 ± 0.07 **	0.44 ± 0.04 **
*age-1*	1.00 ± 0.03	0.62 ± 0.02 **	0.94 ± 0.06
*akt-2*	1.00 ± 0.07	0.73 ± 0.04 **	0.62 ± 0.04 **
*skn-1*	1.00 ± 0.02	2.09 ± 0.18 **	2.37 ± 0.16 **
*hsf-1*	1.00 ± 0.02	2.12 ± 0.10 *	3.22 ± 0.63 **
*daf-16*	1.00 ± 0.15	1.33 ± 0.01 **	1.29 ± 0.05 *
*hsp-16.2*	1.00 ± 0.12	1.62 ± 0.05 *	2.31 ± 0.32 **
*sod-3*	1.00 ± 0.09	2.61 ± 0.06 **	1.45 ± 0.19 **
*gst-4*	1.00 ± 0.09	1.60 ± 0.06 *	2.84 ± 0.29 **
*fil-2*	1.00 ± 0.11	2.99 ± 0.33 **	——
*lips-11*	1.00 ± 0.09	3.28 ± 0.47 **	——
*cyp-35A4*	1.00 ± 0.01	1.52 ± 0.13 **	——
*aex-2*	1.00 ± 0.02	7.68 ± 0.12 **	——

Data are expressed as the mean ± SEM (*n* = 3), * *p* < 0.05, ** *p* < 0.01.

**Table 7:** *C. elegans* strains and genotypes.

Strains	Genotype
N2	*C. elegans* wild isolate
TJ375	gpIs1 [*hsp-16.2p*::GFP]
CF1553	muIs84 [(pAD76) *sod-3p*::GFP + rol-6 (su1006)]
CL2166	dvIs19 [(pAF15) *gst-4p*::GFP::NLS] III

**Table 8:** Primers for qPCR assays.

Gene	Forward (5′→3′)	Reverse (5′→3′)
*daf-2*	TGCTCACCTGTACCTTCTCCTTC	CCTTCAACTCGGACGCCATTC
*daf-16*	AAGCGTGGAACTGTCGTG	GGTTACTCCGCCAAAAGAA
*age-1*	CTGGCATCAAAATCTACAACA	CCAATCGGTTAGGGACATA
*akt-2*	ACTTGCTCTTGGATACCTTCATCAC	GGCTTCTGGGCTTAACCTATTCG
*skn-1*	GCGACGAGACGAGACGATAAAC	GTTCTTGTTGATGCTGATGGTTGAC
*hsf-1*	TCCAACGACTTCCACCTCATCC	AACAACAAATCCTCGGCTCCATC
*hsp-16.2*	AACGCCAATTTGCTCCAGTCTG	CTTCCTTGAACCGCTTCTTTCTTTG
*sod-3*	TGGTGGTGGACACATCAATCATTC	GCACAGGTGGCGATCTTCAAG
*gst-4*	TGGAGAACAATTCGGTCAGTCAATG	AGCAATCACAATATCAGCCCAAGTC
*fil-2*	GAGTATCAATCAGTGGCAAAGT	CAAGTGGGAGCAAACAAGC
*lips-11*	ACGGAGGTATTCTGTGCG	TCAATGTGGAAAAGGGCTA
*cyp-35A4*	ATGGTTCCGGTTTGGATT	AACTCTTTTGATTGCTTCTGC
*aex-2*	TCGGCTCTTGTACCTATTCA	GCCTTTCAGATGCTCGTC
*ß-actin*	GTCATGGTCGGTATGGGACA	TTGTAGAAGGTGTGATGCCAGA

## Data Availability

Data are contained within the article.

## Supplementary Materials

The following supporting information can be downloaded at: https://www.mdpi.com/article/10.3390/molecules30173463/s1, File S1: Certificate of Analysis.

## Abbreviations

The following abbreviations are used in this manuscript.

BCA	Bicinchoninic Acid Assay
CAT	catalase
*C. elegans*	*Caenorhabditis elegans*
CGC	*Caenorhabditis* Genetics Center
IIS	Insulin/Insulin-like Growth Factor-1 Signaling
MET	Metformin
MDA	malondialdehyde
NGM	nematode growth medium
ROS	reactive oxygen species
SOD	superoxide dismutase
