# Supplementary material for: Ginsenoside Re Regulates the Insulin/Insulin-like Growth Factor-1 Signaling Pathway and Mediates Lipid Metabolism to Achieve Anti-Aging Effects in Caenorhabditis elegans
Source: Molecules. 2025 Aug 22;30(17):3463. doi: 10.3390/molecules30173463 (PMC12430152; doi:10.3390/molecules30173463)
Supplement: Supplementary file 1 [file molecules-30-03463-s001.zip › molecules-3770541 - Supplementary Material S1-English version.pdf]

植标化纯生物
Purechem—Standard

**Description**	Ginsenoside Rb1	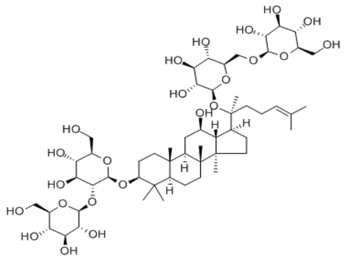
**CAS No.**	41753-43-9	
**MF**	C54H92023	
**MW**	1109.29	

TEST ITEM AND RESULT

Item	Standard	Result	REMARKS
**Appearance**	**powder**	**Conforms**	
**Loss on drying**	**≤ 2.0%**	**Conforms**	
**Assay by HPLC**	**≥ 98%**	**98.88%**	

ATTENTION

**Storage**	**Keep tightly sealed and store under dry and dark conditions. Recommended storage temperature: below 4 。C, special varieties below -20。 C.**
**Warranty**	**Two years**
**Usage**	**Because some compounds may change at room temperature after dissolved in solvents, please use the dissolved sample early. Chromatographic pure reagents are recommended to dissolve samples for HPLC analysis.**
**Note**	**In case of quality problem, please contact us within 15 days after receiving the products.**

办公地址：成都市航空港空港国际城一期
生产地址：四川成都天府新区乐至西郊工业园
电话：028-84248411 传真：028-61551164
网址：https://www.herbsubstance.com

色谱条件：照高效液相色谱法测定，以十八烷基硅烷键合硅胶为填充剂；

流 动 相：乙腈：0.05%磷酸　25:75--50:50　　0--20 分钟
检测波长：203NM

附：HPLC 图谱

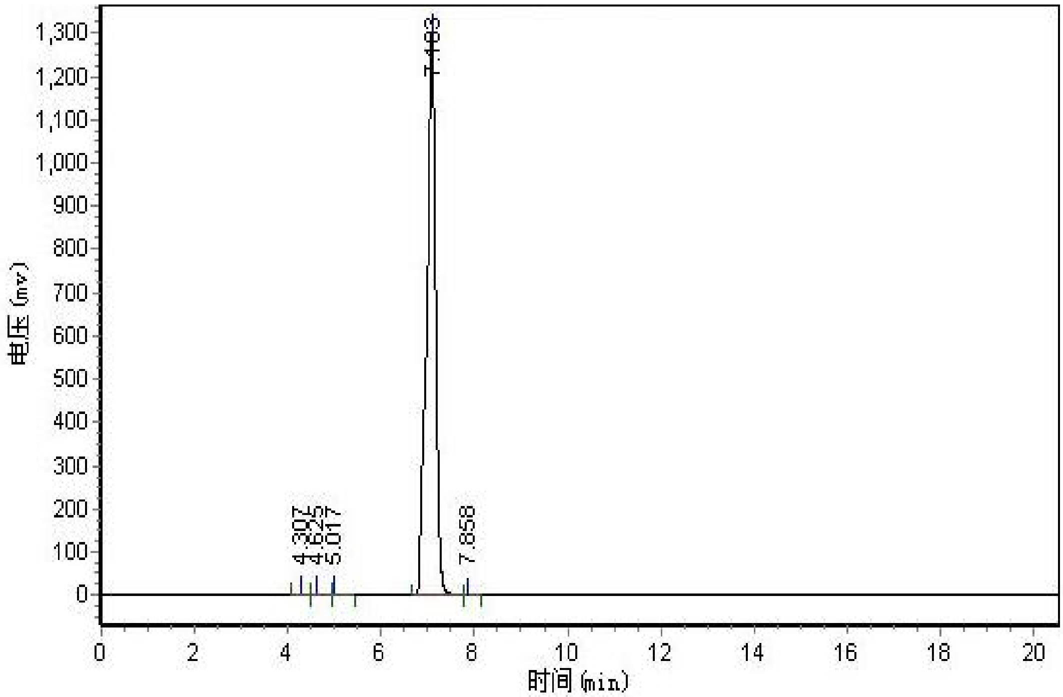

分析结果表

峰号	峰名	保留时间	峰高	峰面积	含量
1		4.307	3893.502	51608.059	0.2908
2		4.625	4231.850	87067.813	0.4905
3		5.017	3645.741	50326.457	0.2835
4		7.103	1299123.625	17551142.000	98.8806
5		7.858	774.972	9689.460	0.0546
总计			1311669.690	17749833.788	100.0000

Description	Ginsenoside Rb2	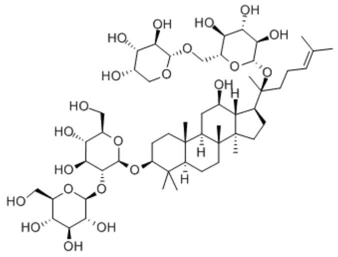
CAS No.	11021-13-9	
MF	C53H90022	
MW	1079.27	

TEST ITEM AND RESULT

Item	Standard	Result	REMARKS
Appearance	powder	Conforms	
Loss on drying	≤ 2.0%	Conforms	
Assay by HPLC	≥ 98%	98.5%	

ATTENTION

Storage	Keep tightly sealed and store under dry and dark conditions. Recommended storage temperature: below 4 。C, special varieties below -20。C.
Warranty	Two years
Usage	Because some compounds may change at room temperature after dissolved in solvents, please use the dissolved sample early. Chromatographic pure reagents are recommended to dissolve samples for HPLC analysis.
Note	In case of quality problem, please contact us within 15 days after receiving the products.

办公地址：成都市航空港空港国际城一期
生产地址：四川成都天府新区乐至西郊工业园
电话：028-84248411 传真：028-61551164
网址：https://www.herbsubstance.com

植标化纯生物
Purechem—Standard

色谱条件：照高效液相色谱法测定，以十八烷基硅烷键合硅胶为填充剂；

流 动 相：乙腈：0.05%磷酸 25:75--50:50 0--20 分钟
检测波长：203NM

附：HPLC 图谱

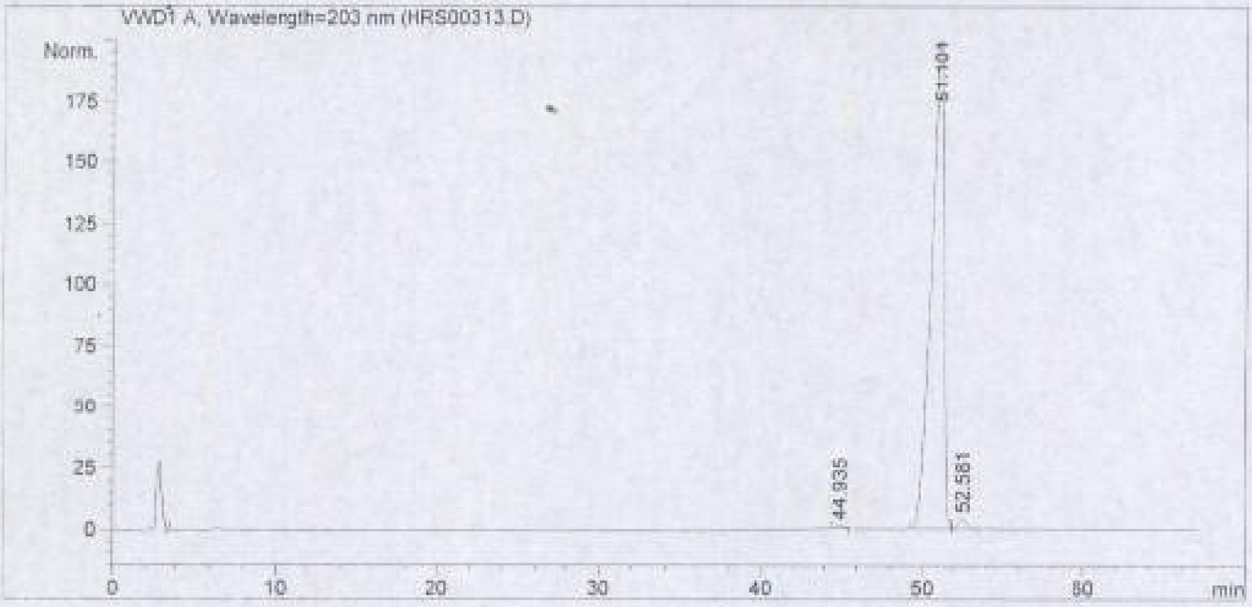

Area Percent Report

Sorted By : Signal
Multiplier : 1.0000
Dilution : 1.0000
Use Multiplier & Dilution Factor with ISTDs

Signal 1: VWD1 A, Wavelength=203 nm

Peak #	RetTime [min]	Type	Width [min]	Area mAU *s	Height [mAU]	Area %
1	44.935	BB	0.4467	35.05943	1.08543	0.2702
2	51.101	BV	0.9338	1.27791e4	216.78085	98.5053
3	52.581	VB	0.5506	158.84383	4.18505	1.2244
Totals :				1.29730e4	222.05134	

附：核磁

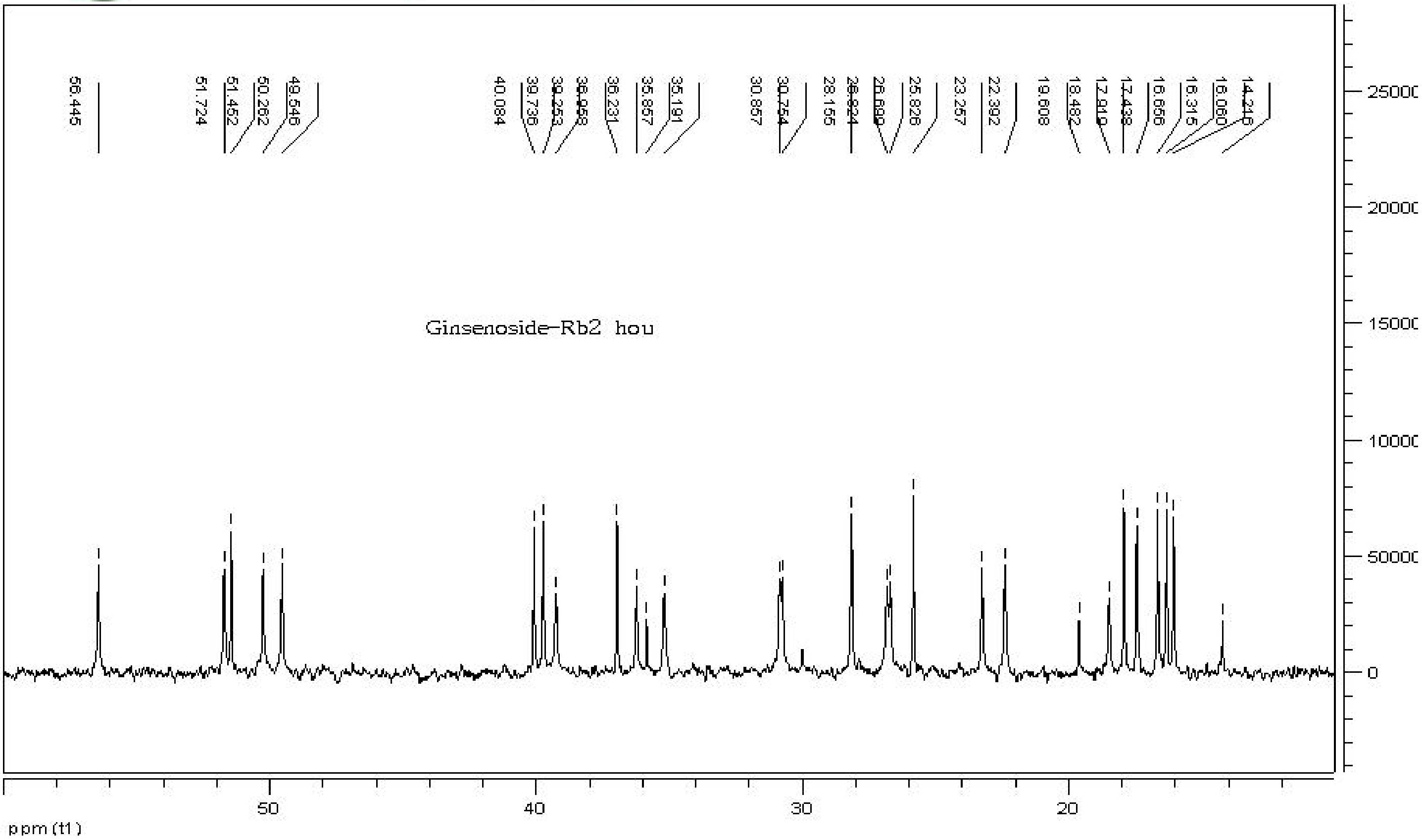

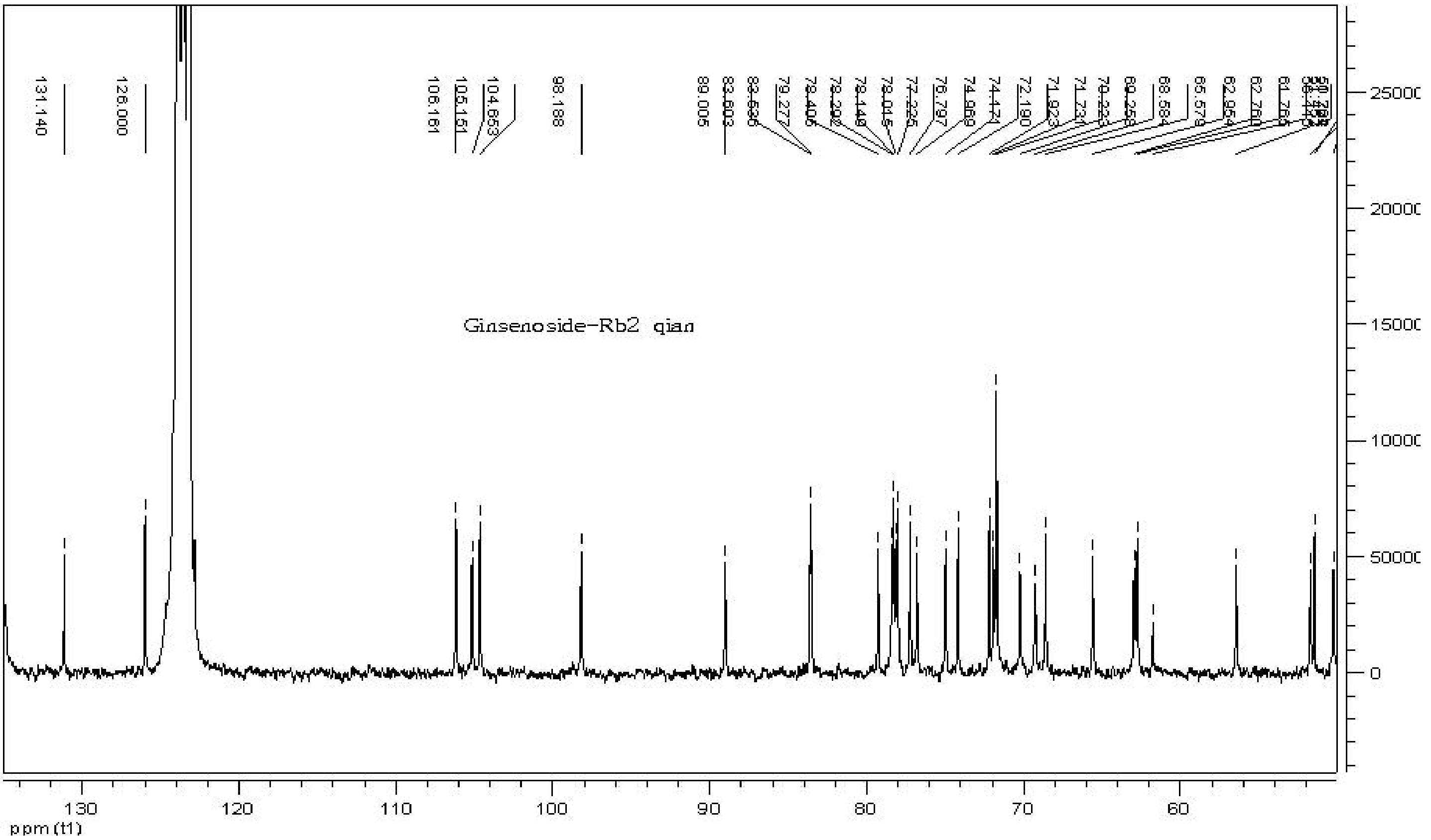

办公地址：成都市航空港空港国际城一期
生产地址：四川成都天府新区乐至西郊工业园
电话：028-84248411 传真：028-61551164
网址：https://www.herbsubstance.com

**Description**	Ginsenoside Rb3	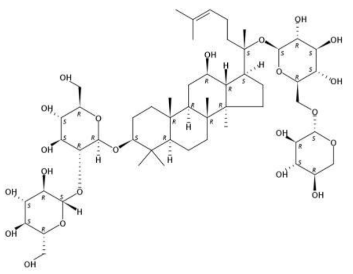
**CAS No.**	68406-26-8	
**MF**	C53H90O22	
**MW**	1079.27	

TEST ITEM AND RESULT

Item	Standard	Result	REMARKS
**Appearance**	**powder**	**Conforms**	
**Loss on drying**	**≤ 2.0%**	**Conforms**	
**Assay by HPLC**	**≥ 98%**	**99.29%**	

ATTENTION

**Storage**	**Keep tightly sealed and store under dry and dark conditions. Recommended storage temperature: below 4 。C, special varieties below -20。 C.**
**Warranty**	**Two years**
**Usage**	**Because some compounds may change at room temperature after dissolved in solvents, please use the dissolved sample early. Chromatographic pure reagents are recommended to dissolve samples for HPLC analysis.**
**Note**	**In case of quality problem, please contact us within 15 days after receiving the products.**

办公地址：成都市航空港空港国际城一期
生产地址：四川成都天府新区乐至西郊工业园
电话：028-84248411 传真：028-61551164
网址：https://www.herbsubstance.com

色谱条件：照高效液相色谱法测定，以十八烷基硅烷键合硅胶为填充剂；

Column:SVEA C18 Opal 4.6*150 mm,5μm;
Column temperature: 35℃;
DetectionMode:UV203 nm;
Flow Rate:1.0ml/min;
Sample dissolution:Methanol;
Mobile Phase:A-Acetonitrile ,B-0.1% Phosphoric acid in water;
Gradient elution:A,29%-39%,15min,39%-90%,3min.

附：HPLC 图谱

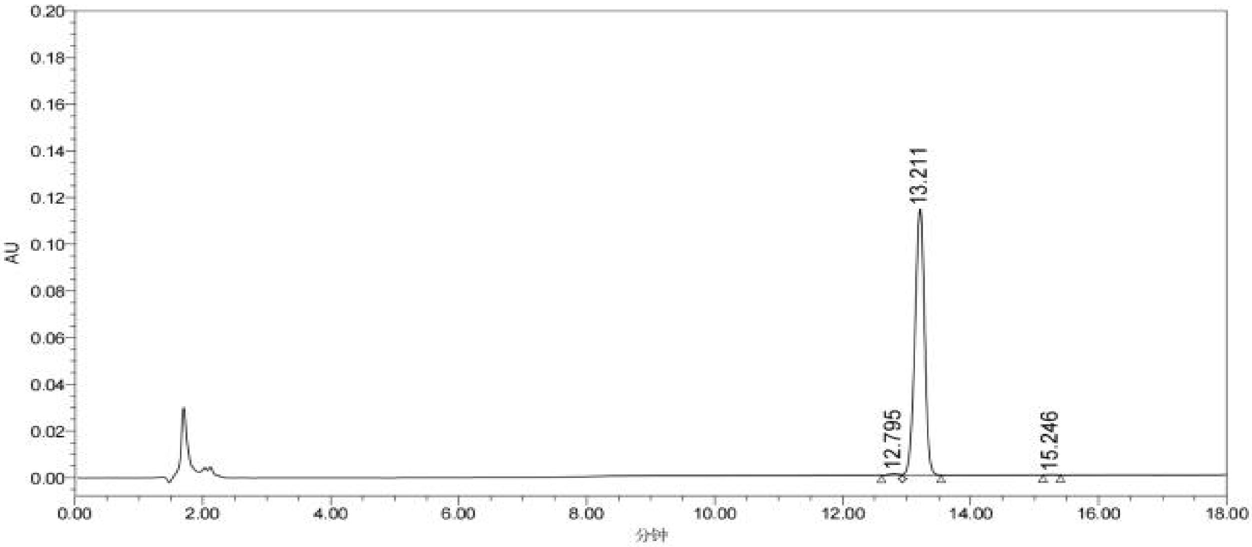

色谱峰结果

	名称	保留时间	面积	峰高	% 面积
1		12.795	7254	737	0.61
2		13.211	1183066	113986	99.29
3		15.246	1186	121	0.10

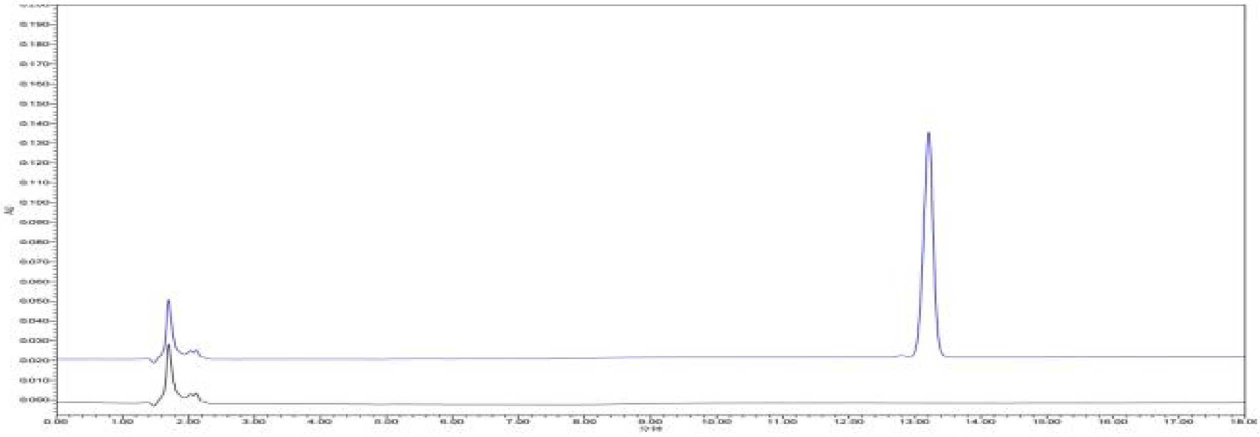

附：核磁

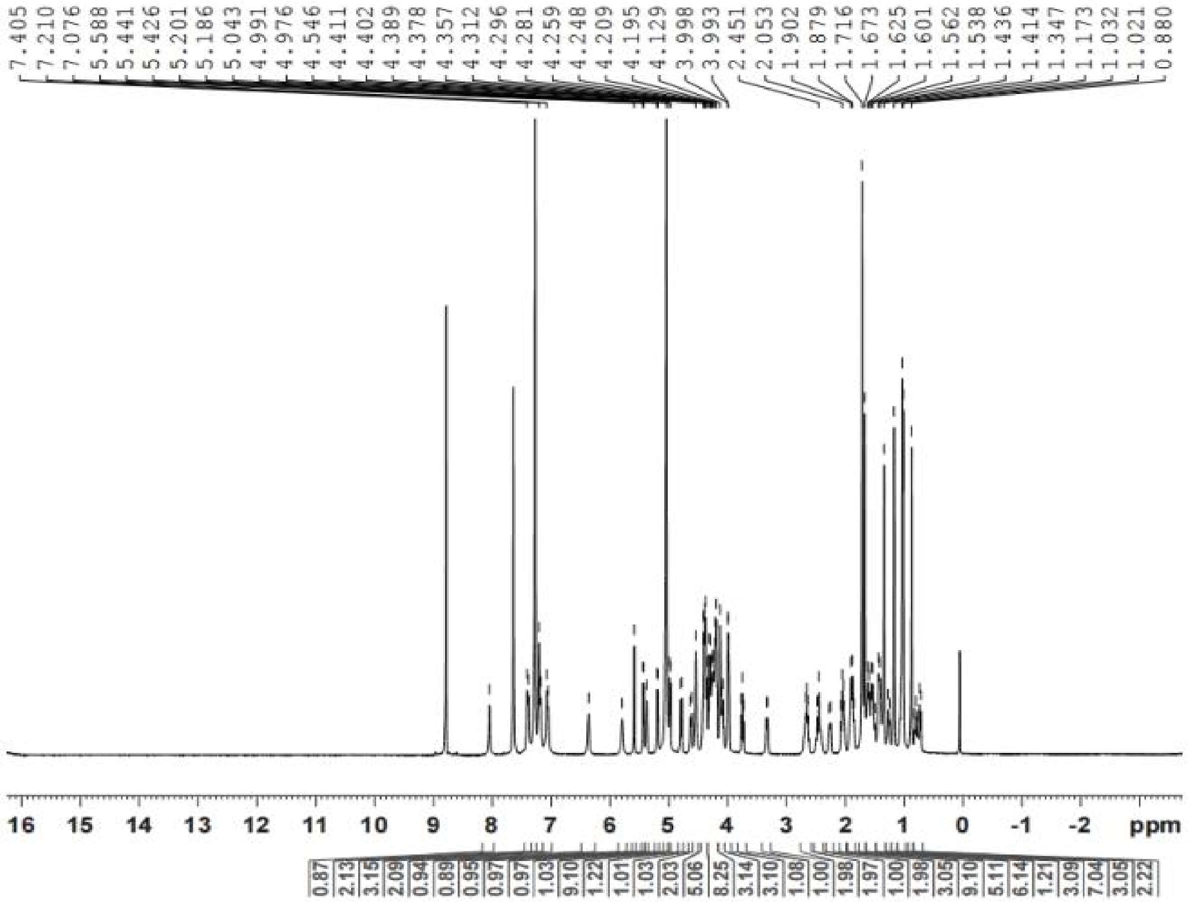
7.405
7.210
7.076
5.588
5.441
5.426
5.201
5.186
5.043
4.991
4.976
4.546
4.411
4.402
4.389
4.378
4.357
4.312
4.296
4.281
4.259
4.248
4.209
4.195
4.129
3.998
3.993
2.451
2.053
1.902
1.879
1.716
1.673
1.625
1.601
1.562
1.538
1.436
1.414
1.347
1.173
1.032
1.021
0.880
16 15 14 13 12 11 10 9 8 7 6 5 4 3 2 1 0 -1 -2 ppm
0.87 2.13 3.15 2.09 0.94 0.89 0.95 0.97 0.97 1.03 9.10 1.22 1.01 1.03 2.03 5.06 8.25 3.14 3.10 1.08 1.00 1.98 1.97 1.00 1.98 3.05 9.10 5.11 6.14 1.21 3.09 7.04 3.05 2.22

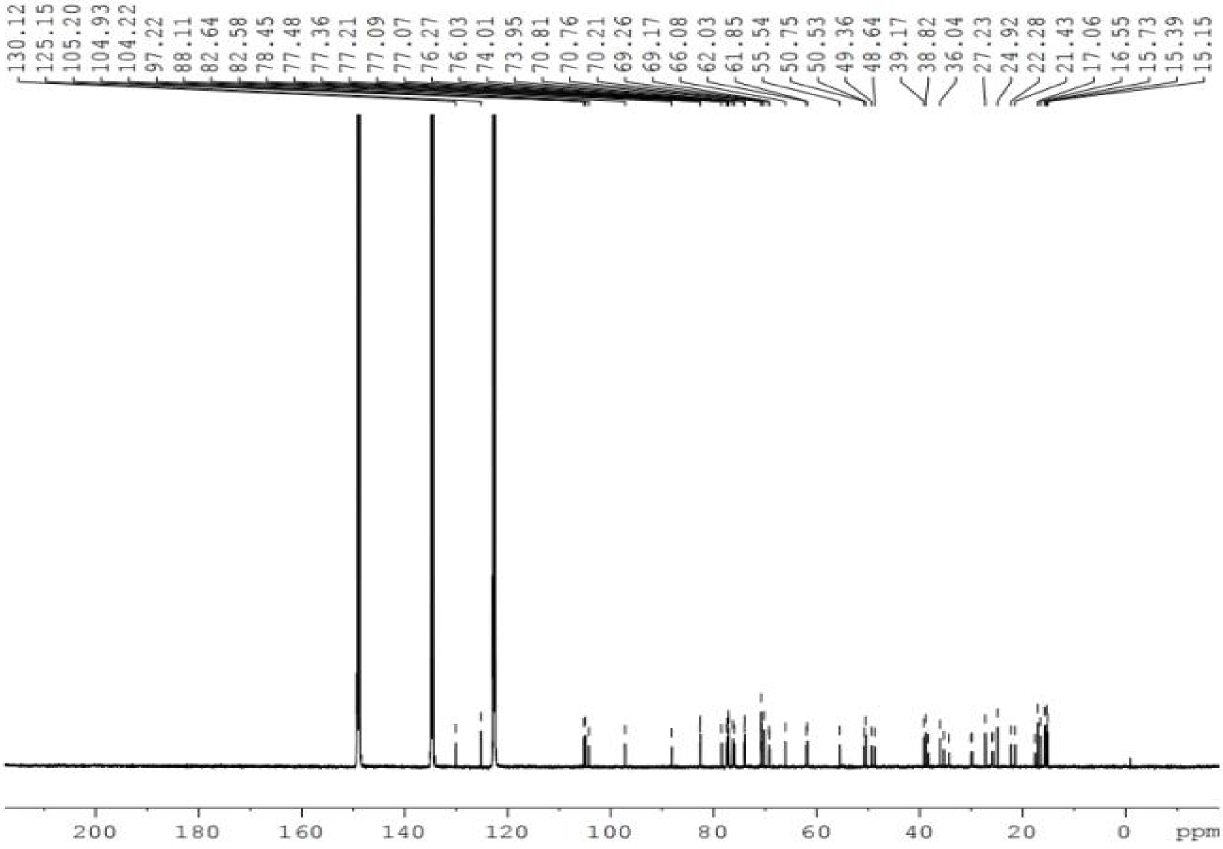
130.12
125.15
105.20
104.93
104.22
97.22
88.11
82.64
82.58
78.45
77.48
77.36
77.21
77.09
77.07
76.27
76.03
74.01
73.95
70.81
70.76
70.21
69.26
69.17
66.08
62.03
61.85
55.54
50.75
50.53
49.36
48.64
39.17
38.82
36.04
27.23
24.92
22.28
21.43
17.06
16.55
15.73
15.39
15.15
200 180 160 140 120 100 80 60 40 20 0 ppm

附：质谱

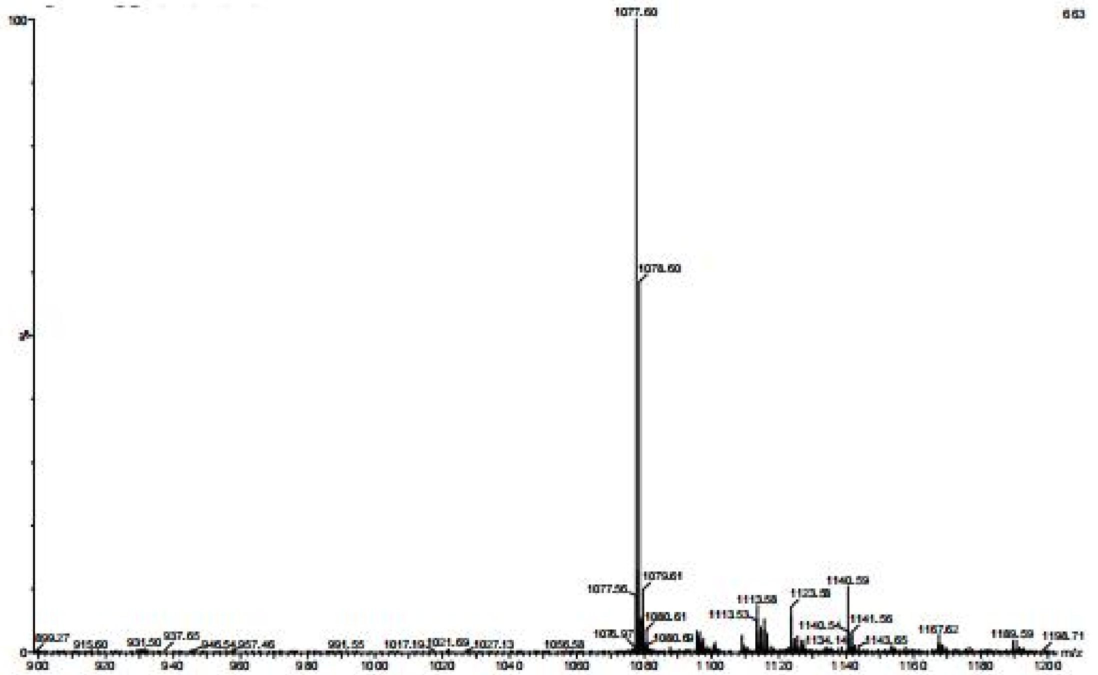
1077.60
1078.60
1079.61
1140.59
m/z

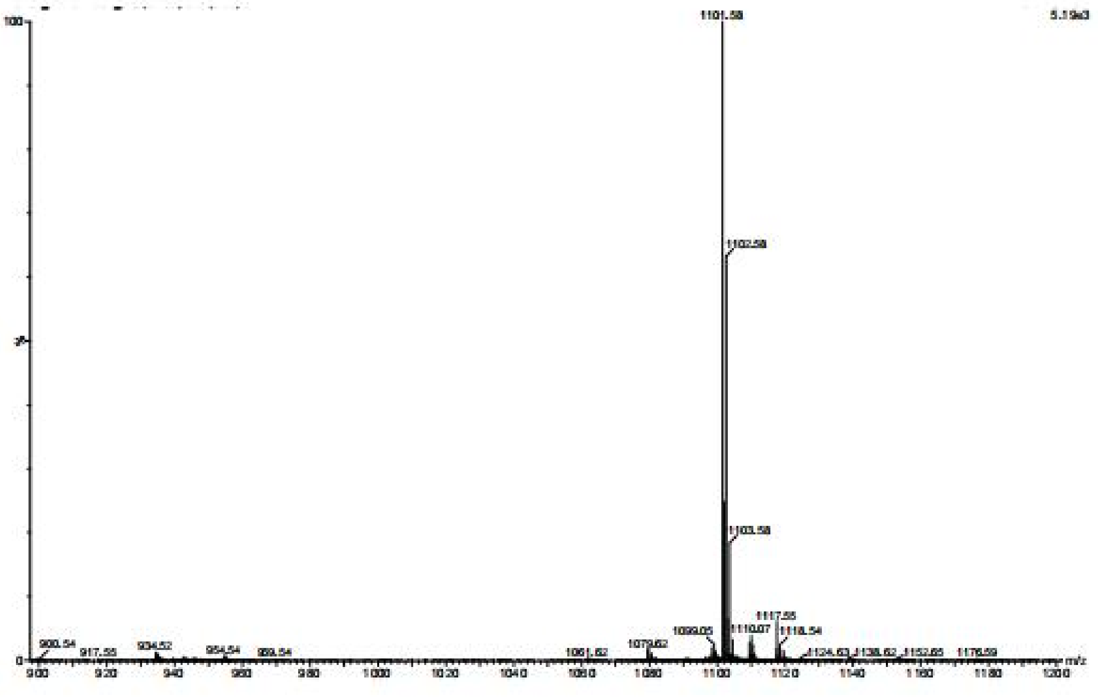
1101.58
1102.58
1103.58
1117.55
m/z

**Description**	**Ginsenoside RC**	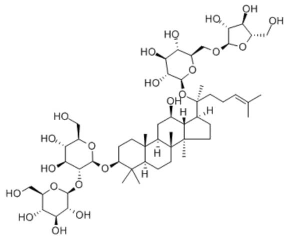
**CAS No.**	11021-14-0	
**MF**	C53H90022	
**MW**	1079.27	

TEST ITEM AND RESULT

Item	Standard	Result	REMARKS
Appearance	powder	Conforms	
Loss on drying	≤ 2.0%	Conforms	
Assay by HPLC	≥ 98%	99.37%	

ATTENTION

**Storage**	**Keep tightly sealed and store under dry and dark conditions. Recommended storage temperature: below 4 。C, special varieties below -20。 C.**
**Warranty**	**Two years**
**Usage**	**Because some compounds may change at room temperature after dissolved in solvents, please use the dissolved sample early. Chromatographic pure reagents are recommended to dissolve samples for HPLC analysis.**
**Note**	**In case of quality problem, please contact us within 15 days after receiving the products.**

色谱条件：照高效液相色谱法测定，以十八烷基硅烷键合硅胶为填充剂；

洗脱剂：乙腈/水=35/65，40min
流　速：0.8ml/min
色谱柱：依利特 Sinachrom　ODS　BP,　5um,250*4.6mm
柱　温：40° C

附：HPLC 图谱

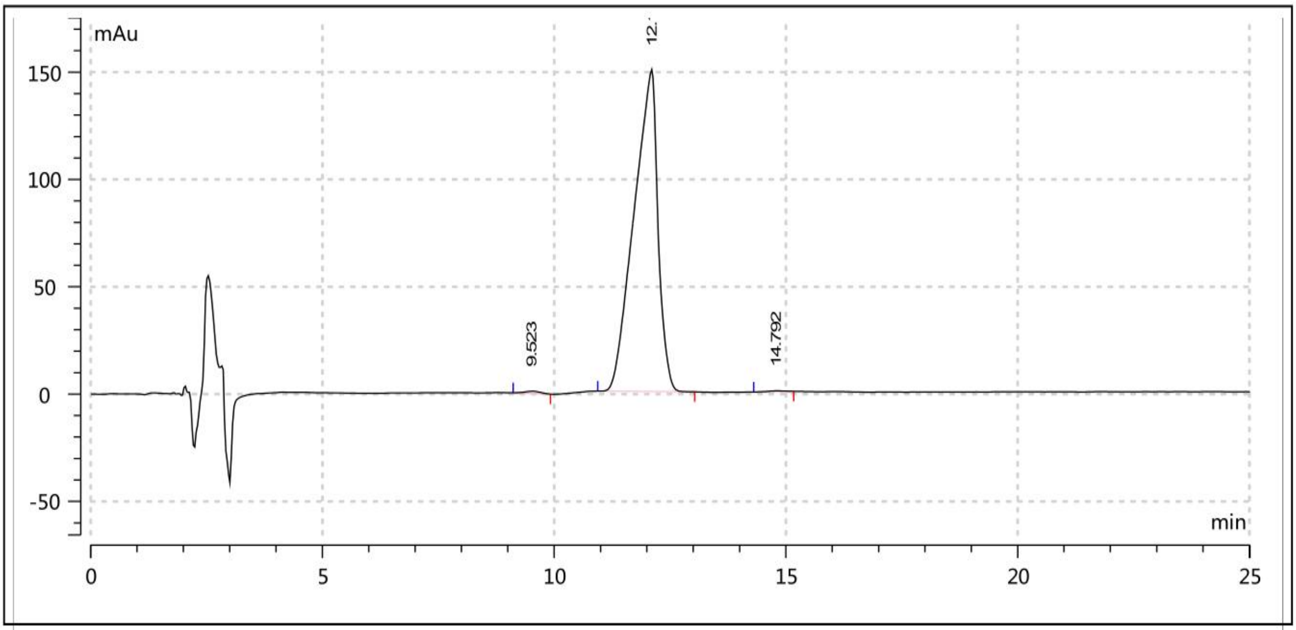

峰列表

No	名称	保留时间 (min)	峰面积 (mAu*s)	峰宽 (min)	半峰宽 (min)	峰高 (mAu)	面积百分比 (%)
1	N.A.	9.523	24.17637	0.515	0.380	1.034	0.446
2	N.A.	12.103	5391.17136	1.015	0.564	150.066	99.378
3	N.A.	14.792	9.58858	0.642	0.397	0.383	0.177
4	总计						

植标化纯生物
Purechem—Standard

氢谱

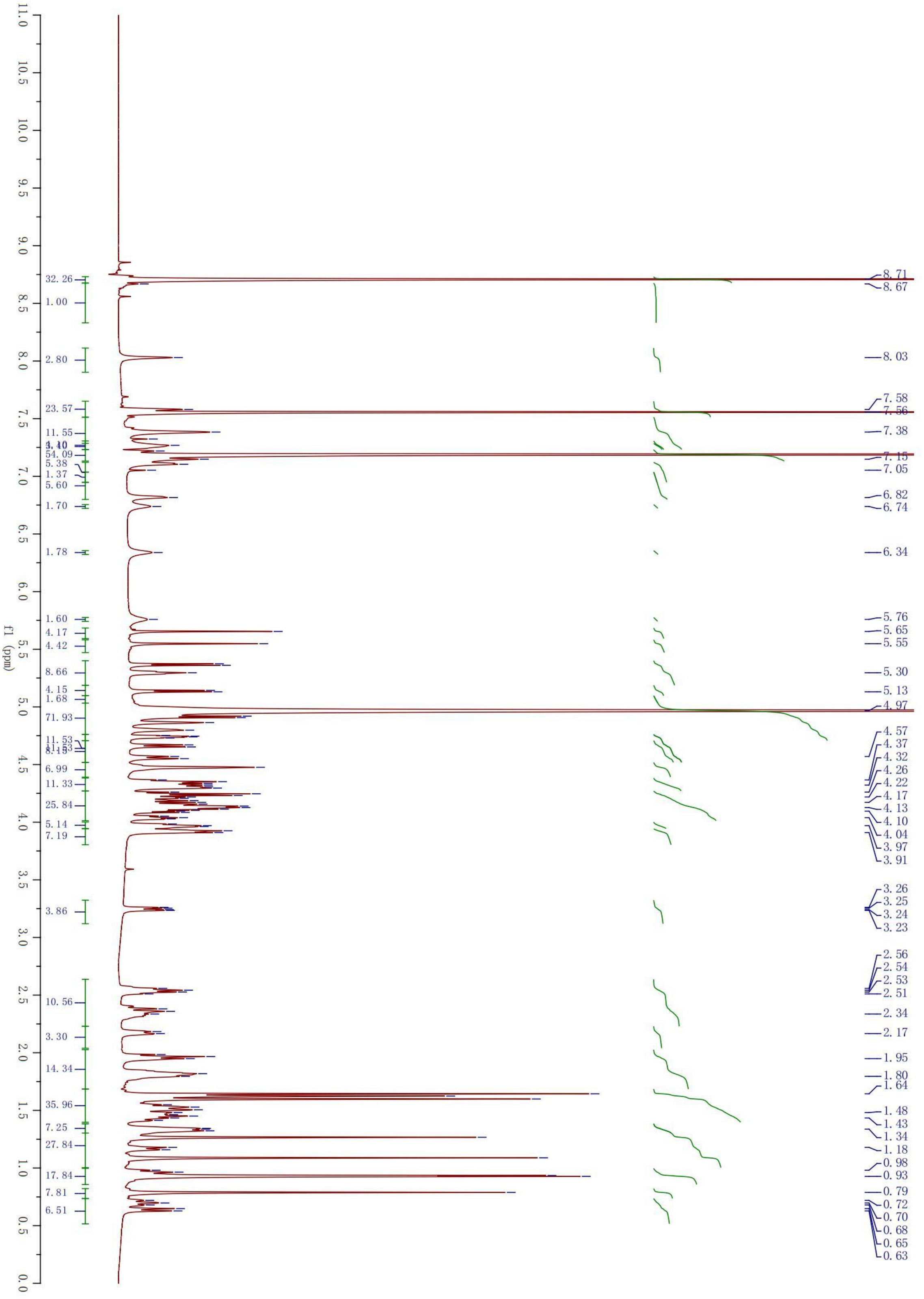

碳谱

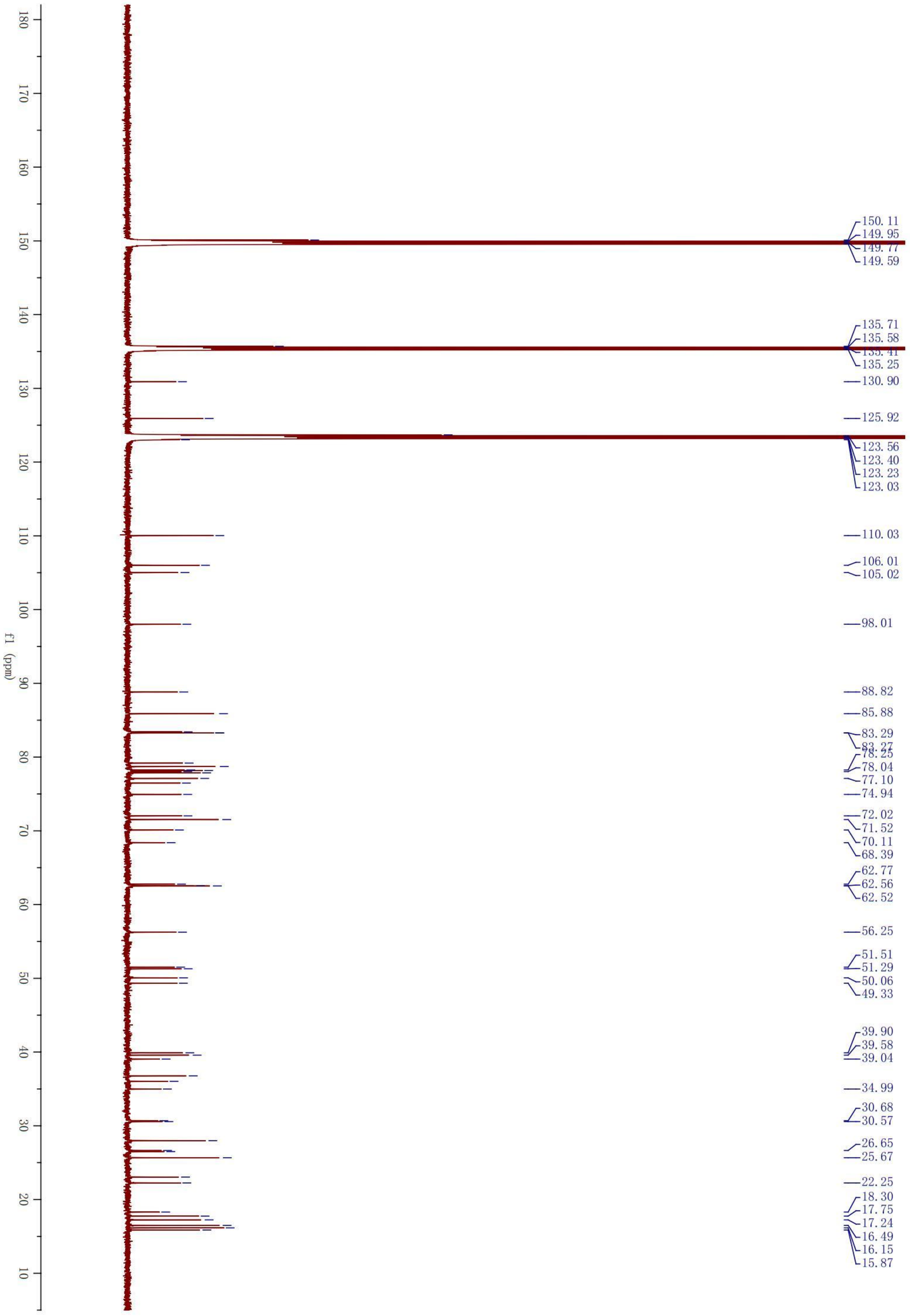

f1 (ppm)
180
170
160
150
140
130
120
110
100
90
80
70
60
50
40
30
20
10
150.11
149.95
149.77
149.59
135.71
135.58
135.41
135.25
130.90
125.92
123.56
123.40
123.23
123.03
110.03
106.01
105.02
98.01
88.82
85.88
83.29
83.27
78.25
78.04
77.10
74.94
72.02
71.52
70.11
68.39
62.77
62.56
62.52
56.25
51.51
51.29
50.06
49.33
39.90
39.58
39.04
34.99
30.68
30.57
26.65
25.67
22.25
18.30
17.75
17.24
16.49
16.15
15.87

植标化纯生物
Purechem—Standard

Description	Ginsenoside RD	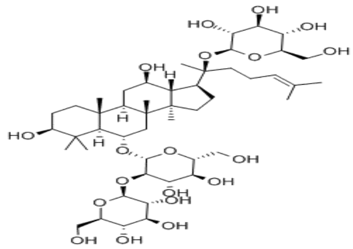
CAS No.	52705-93-8	
MF	C48H82018	
MW	947.15	

TEST ITEM AND RESULT

Item	Standard	Result	REMARKS
Appearance	powder	Conforms	
Loss on drying	≤ 2.0%	Conforms	
Assay by HPLC	≥ 98%	98.54%	

ATTENTION

Storage	Keep tightly sealed and store under dry and dark conditions. Recommended storage temperature: below 4 。C, special varieties below -20。 C.
Warranty	Two years
Usage	Because some compounds may change at room temperature after dissolved in solvents, please use the dissolved sample early. Chromatographic pure reagents are recommended to dissolve samples for HPLC analysis.
Note	In case of quality problem, please contact us within 15 days after receiving the products.

办公地址：成都市航空港空港国际城一期
生产地址：四川成都天府新区乐至西郊工业园
电话：028-84248411 传真：028-61551164
网址：https://www.herbsubstance.com

色谱条件：照高效液相色谱法测定，以十八烷基硅烷键合硅胶为填充剂；

流 动 相：乙腈-水=32-68 为流动相，等度
检测波长：203nm，
流　　速 1mL/min

附：HPLC 图谱

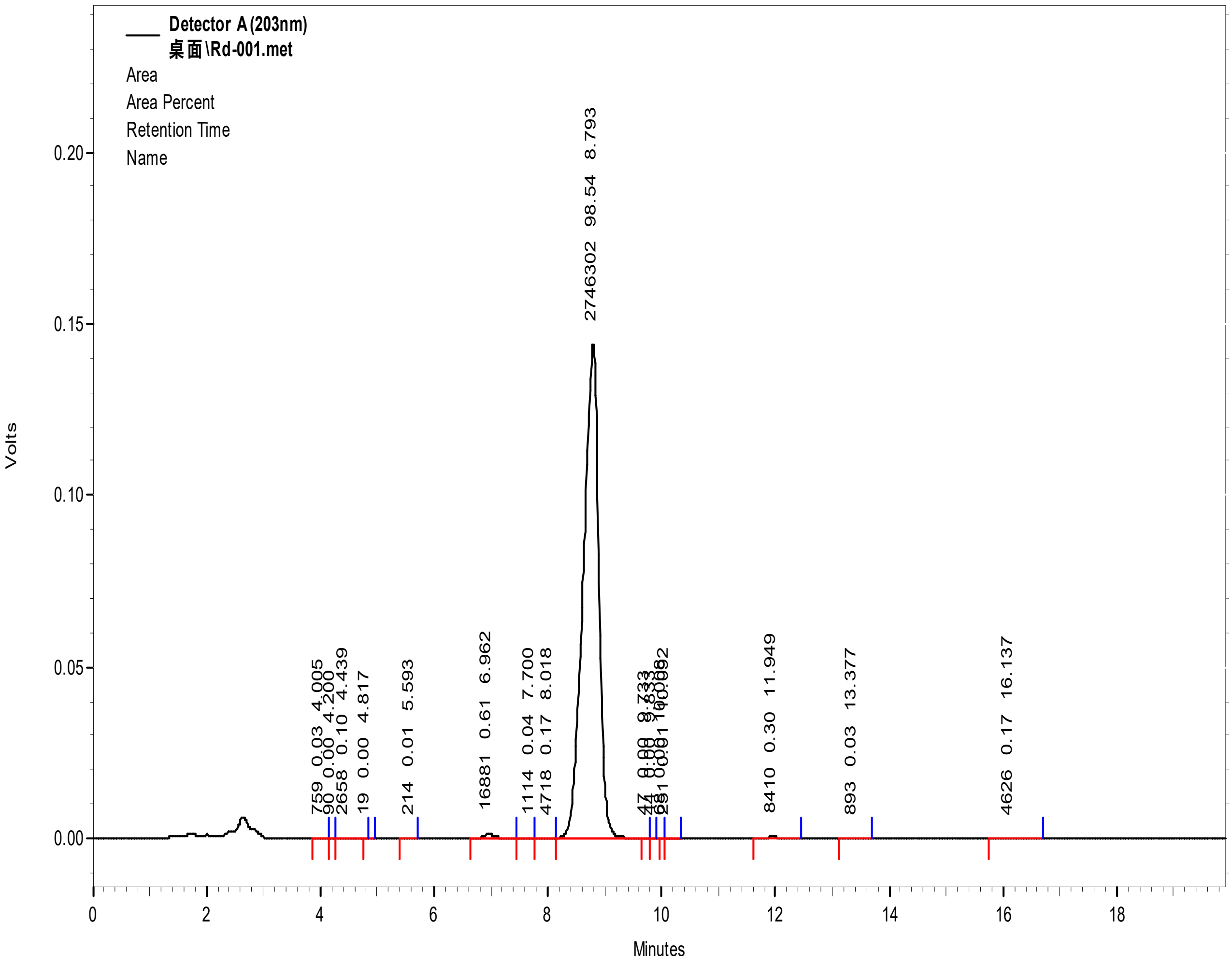

Description	Ginsenoside Re	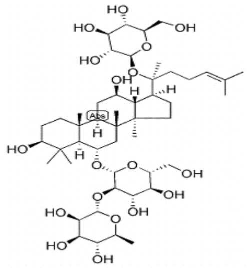
CAS No.	52286-59-6	
MF	C48H82018	
MW	947.15	

TEST ITEM AND RESULT

Item	Standard	Result	REMARKS
Appearance	powder	Conforms	
Loss on drying	≤ 2.0%	Conforms	
Assay by HPLC	≥ 98%	99.08%	

ATTENTION

Storage	Keep tightly sealed and store under dry and dark conditions. Recommended storage temperature: below 4 。C, special varieties below -20。 C.
Warranty	Two years
Usage	Because some compounds may change at room temperature after dissolved in solvents, please use the dissolved sample early. Chromatographic pure reagents are recommended to dissolve samples for HPLC analysis.
Note	In case of quality problem, please contact us within 15 days after receiving the products.

色谱条件：照高效液相色谱法测定，以十八烷基硅烷键合硅胶为填充剂；

检测波长:254nm
流 动 相：乙腈：0.1%磷酸 比例 22:78

附：HPLC 图谱

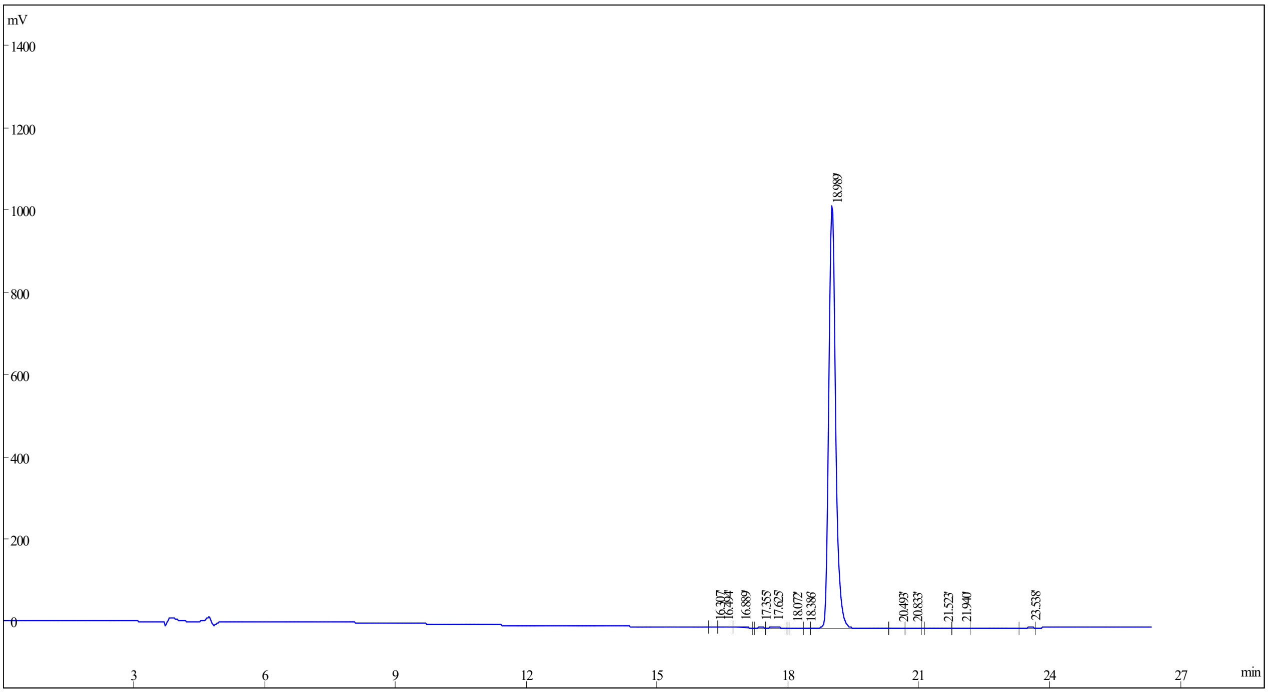

序号	保留时间	浓度	峰面积	峰高
1	16.307	0.007762	892	145
2	16.494	0.06023	6919	771
3	16.889	0.1695	19467	1996
4	17.355	0.0584	6709	938
5	17.625	0.1741	19997	1338
6	18.072	0.01283	1474	74
7	18.386	0.003608	414	57
8	18.989	99.08	11381793	1041393
9	20.493	0.08769	10074	1021
10	20.833	0.05544	6369	699
11	21.523	0.1578	18123	1636
12	21.940	0.06973	8010	812
13	23.538	0.06139	7052	582
总计		100	11487293	1051462

办公地址：成都市航空港空港国际城一期
生产地址：四川成都天府新区乐至西郊工业园
电话：028-84248411 传真：028-61551164
网址：https://www.herbsubstance.com

**Description**	Ginsenoside Rg1	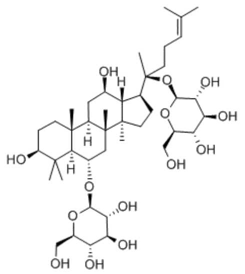
**CAS No.**	22427-39-0	
**MF**	C42H72014	
**MW**	801.01	

TEST ITEM AND RESULT

Item	Standard	Result	REMARKS
Appearance	powder	Conforms	
Loss on drying	≤ 2.0%	Conforms	
Assay by HPLC	≥ 98%	98.59%	

ATTENTION

**Storage**	**Keep tightly sealed and store under dry and dark conditions. Recommended storage temperature: below 4 。C, special varieties below -20。 C.**
**Warranty**	**Two years**
**Usage**	**Because some compounds may change at room temperature after dissolved in solvents, please use the dissolved sample early. Chromatographic pure reagents are recommended to dissolve samples for HPLC analysis.**
**Note**	**In case of quality problem, please contact us within 15 days after receiving the products.**

办公地址：成都市航空港空港国际城一期
生产地址：四川成都天府新区乐至西郊工业园
电话：028-84248411 传真：028-61551164
网址：https://www.herbsubstance.com

色谱条件：照高效液相色谱法测定，以十八烷基硅烷键合硅胶为填充剂；

检测波长: 203nm
流 动 相：乙腈-水=20:80

附：HPLC 图谱

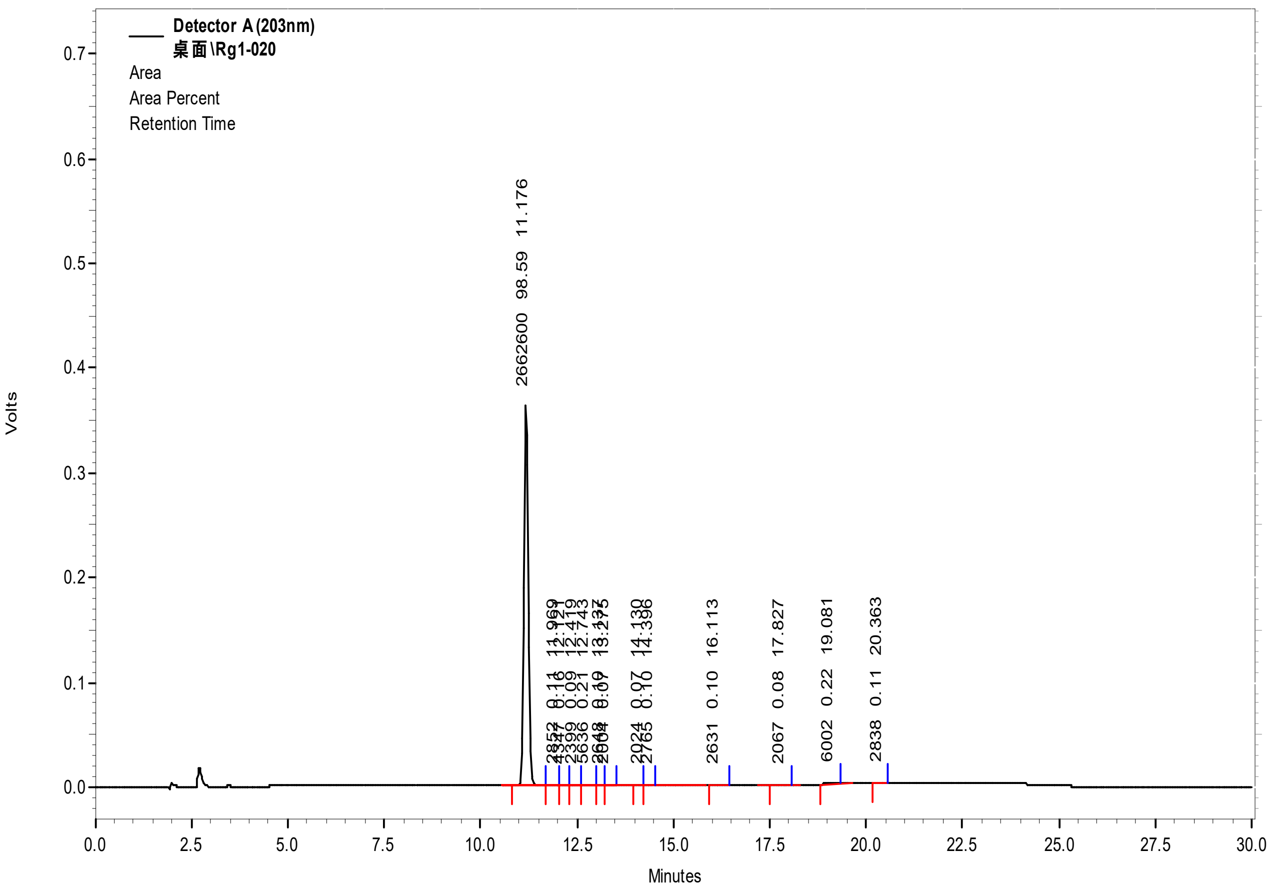

**Description**	Ginsenoside Rg2	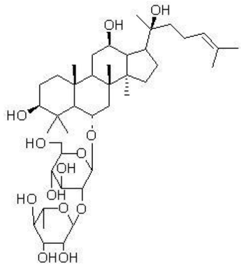
**CAS No.**	52286-74-5	
**MF**	C42H72013	
**MW**	785.01	

TEST ITEM AND RESULT

Item	Standard	Result	REMARKS
**Appearance**	**powder**	**Conforms**	
**Loss on drying**	**≤ 2.0%**	**Conforms**	
**Assay by HPLC**	**≥ 98%**	**99.47%**	

ATTENTION

**Storage**	**Keep tightly sealed and store under dry and dark conditions. Recommended storage temperature: below 4 。C, special varieties below -20。 C.**
**Warranty**	**Two years**
**Usage**	**Because some compounds may change at room temperature after dissolved in solvents, please use the dissolved sample early. Chromatographic pure reagents are recommended to dissolve samples for HPLC analysis.**
**Note**	**In case of quality problem, please contact us within 15 days after receiving the products.**

办公地址：成都市航空港空港国际城一期
生产地址：四川成都天府新区乐至西郊工业园
电话：028-84248411 传真：028-61551164
网址：https://www.herbsubstance.com

色谱条件：照高效液相色谱法测定，以十八烷基硅烷键合硅胶为填充剂；

*流动相：*65%甲醇水
色谱柱： 纳微 ChromCore C18，3u，3*150mm
*流速：*0.35ml/min
*波长：*203nm
溶解溶剂： 甲醇

附：HPLC 图谱

色谱图

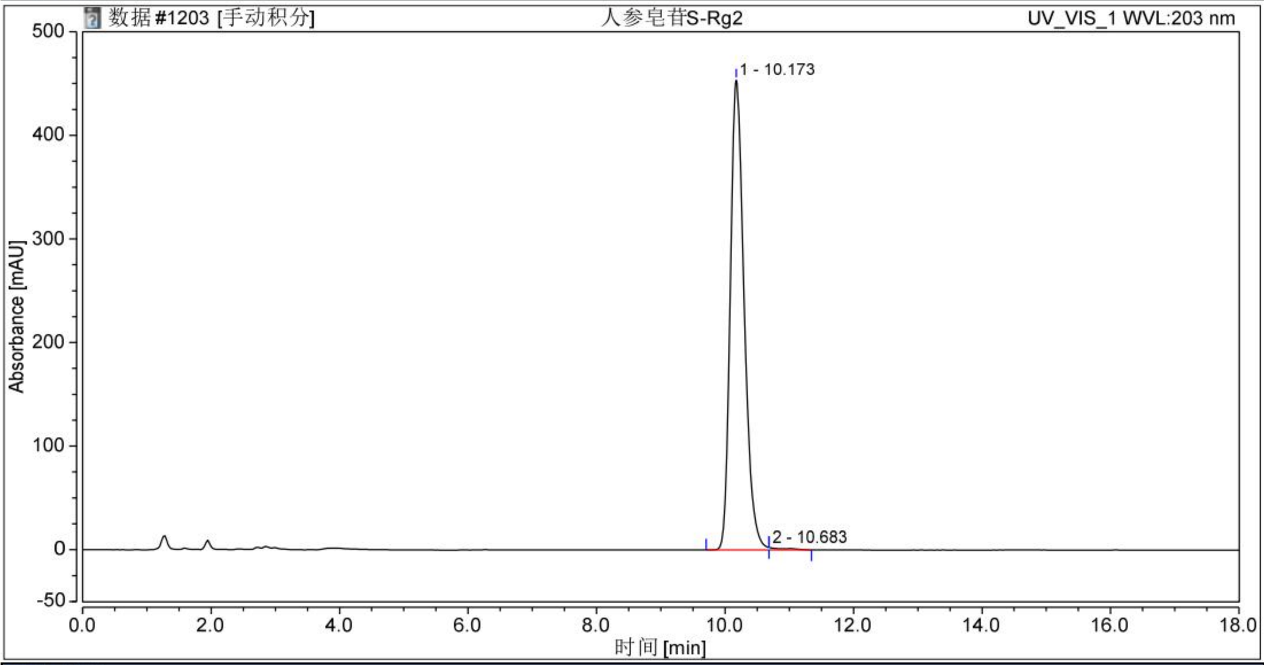

积分结果

序号	峰名称	保留时间 min	峰面积 mAU*min	峰高 mAU	相对峰面积 %	相对峰高 %	样品量 n.a.
1		10.173	113.193	453.428	99.47	99.49	n.a.
2		10.683	0.598	2.335	0.53	0.51	n.a.
总和:			113.791	455.763	100.00	100.00	

Description	Ginsenoside Rg3	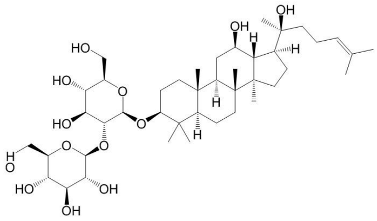
CAS No.	14197-60-5	
MF	C42H72013	
MW	785.025	

TEST ITEM AND RESULT

Item	Standard	Result	REMARKS
Appearance	powder	Conforms	
Loss on drying	≤ 2.0%	Conforms	
Assay by HPLC	≥ 98%	99.83%	

ATTENTION

Storage	Keep tightly sealed and store under dry and dark conditions. Recommended storage temperature: below 4 。C, special varieties below -20。 C.
Warranty	Two years
Usage	Because some compounds may change at room temperature after dissolved in solvents, please use the dissolved sample early. Chromatographic pure reagents are recommended to dissolve samples for HPLC analysis.
Note	In case of quality problem, please contact us within 15 days after receiving the products.

色谱条件：照高效液相色谱法测定，以十八烷基硅烷键合硅胶为填充剂；

Column: Thermo BDS Hypersil C18 4.6*100mm,2.4μm;
Column temperature: 30℃;
Detection Mode: ELSD;
Flow Rate: 0.8ml/min;
Sample dissolution: Methanol;
Mobile Phase: **A,** Acetonitrile **B,** Water ；
Gradient elution: A, 35%-55%,15min-80%,10min.

附：HPLC 图谱

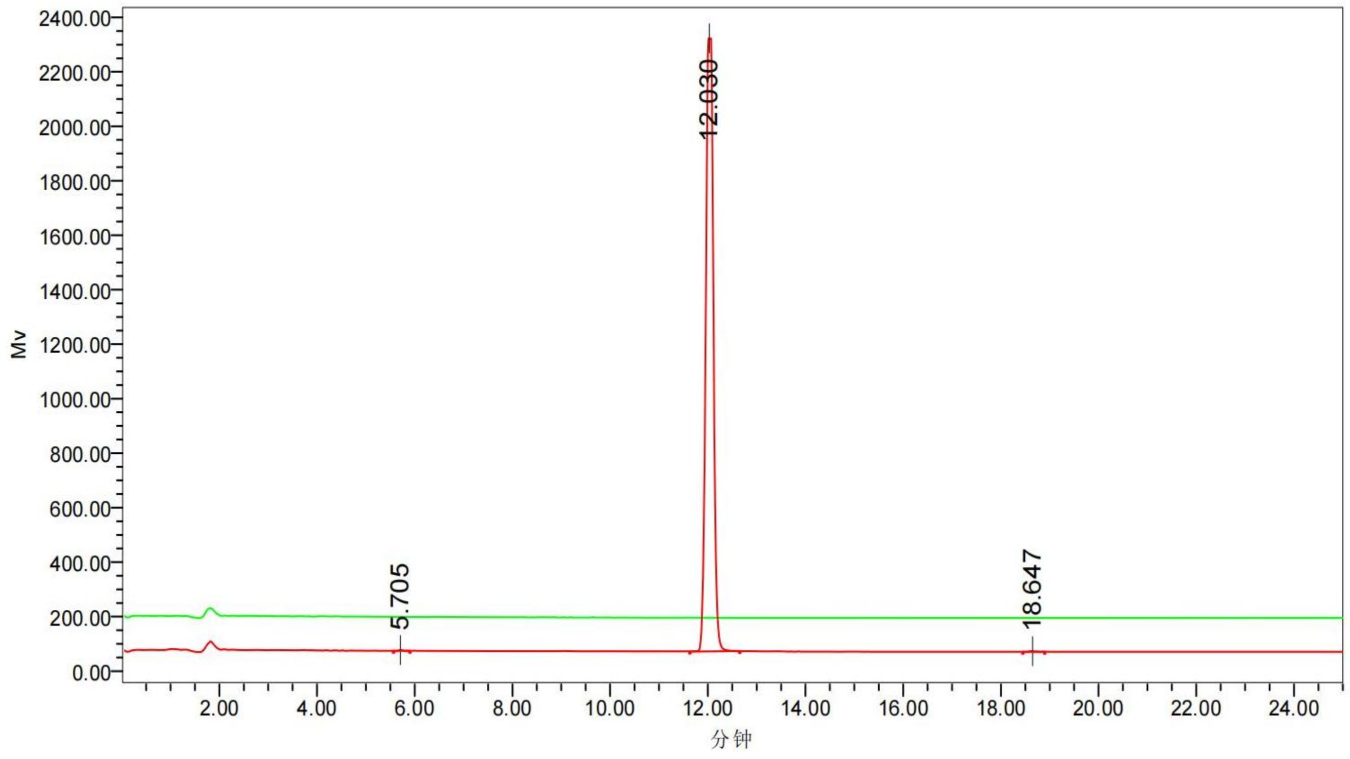

Peak Results

	Name	RT	Area	% Area	USP Plate Count	USP Resolution
1		5.705	17586	0.07	23620.68	
2		12.030	24328333	99.83	35262.98	30.41
3		18.647	24439	0.10	80174.63	24.39
4						

附：核磁

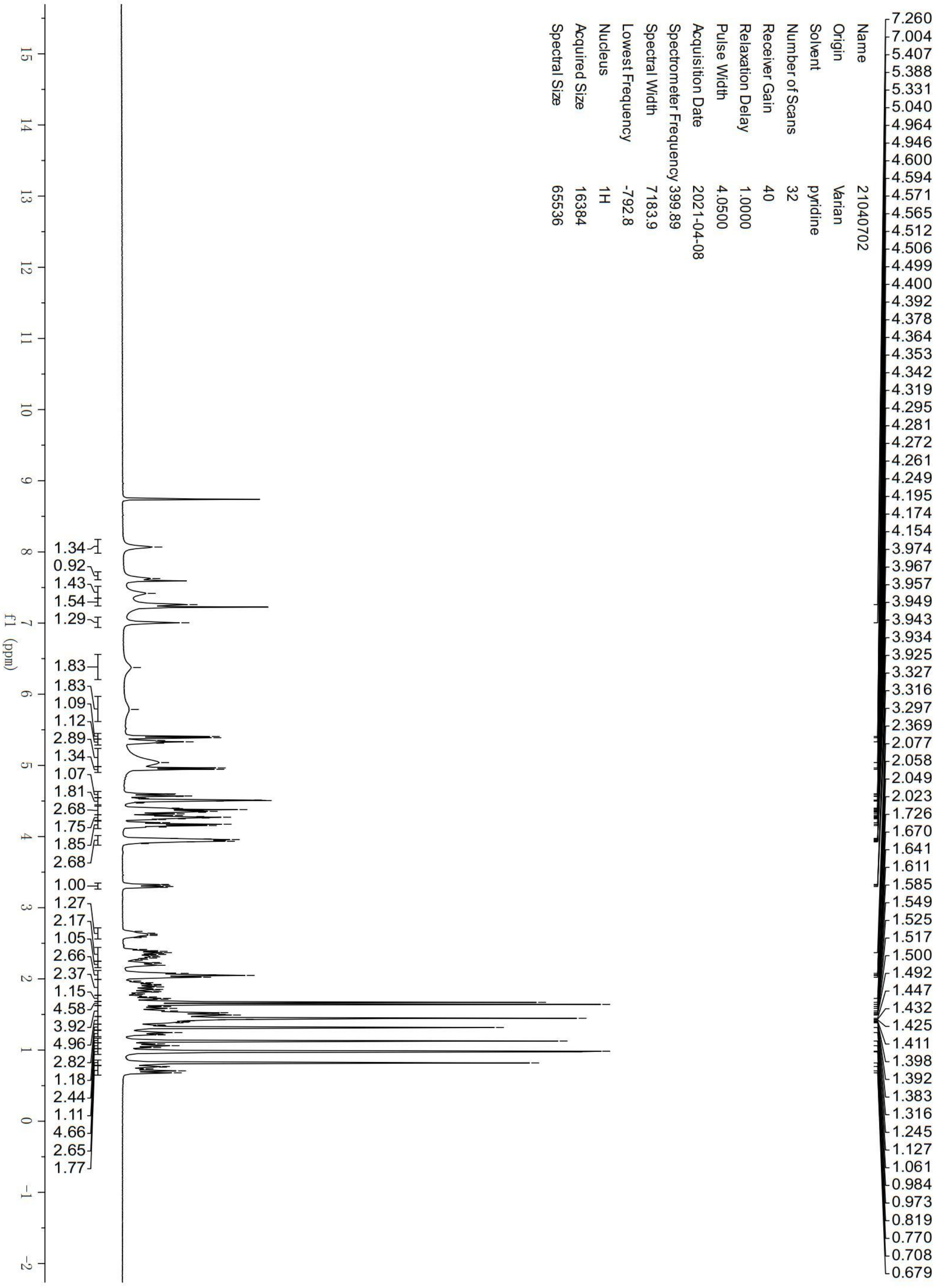
Name 21040702
Origin Varian
Solvent pyridine
Number of Scans 32
Receiver Gain 40
Relaxation Delay 1.0000
Pulse Width 4.0500
Acquisition Date 2021-04-08
Spectrometer Frequency 399.89
Spectral Width 7183.9
Lowest Frequency -792.8
Nucleus 1H
Acquired Size 16384
Spectral Size 65536
7.260 7.004 5.407 5.388 5.331 5.040 4.964 4.946 4.600 4.594 4.571 4.565 4.512 4.506 4.499 4.400 4.392 4.378 4.364 4.353 4.342 4.319 4.295 4.281 4.272 4.261 4.249 4.195 4.174 4.154 3.974 3.967 3.957 3.949 3.943 3.934 3.925 3.327 3.316 3.297 2.369 2.077 2.058 2.049 2.023 1.726 1.670 1.641 1.611 1.585 1.549 1.525 1.517 1.500 1.492 1.447 1.432 1.425 1.411 1.398 1.392 1.383 1.316 1.245 1.127 1.061 0.984 0.973 0.819 0.770 0.708 0.679
1.34 0.92 1.43 1.54 1.29 1.83 1.83 1.09 1.12 2.89 1.34 1.07 1.81 2.68 1.75 1.85 2.68 1.00 1.27 2.17 1.05 2.66 2.37 1.15 4.58 3.92 4.96 2.82 1.18 2.44 1.11 4.66 2.65 1.77
f1 (ppm)
15 14 13 12 11 10 9 8 7 6 5 4 3 2 1 0 -1 -2

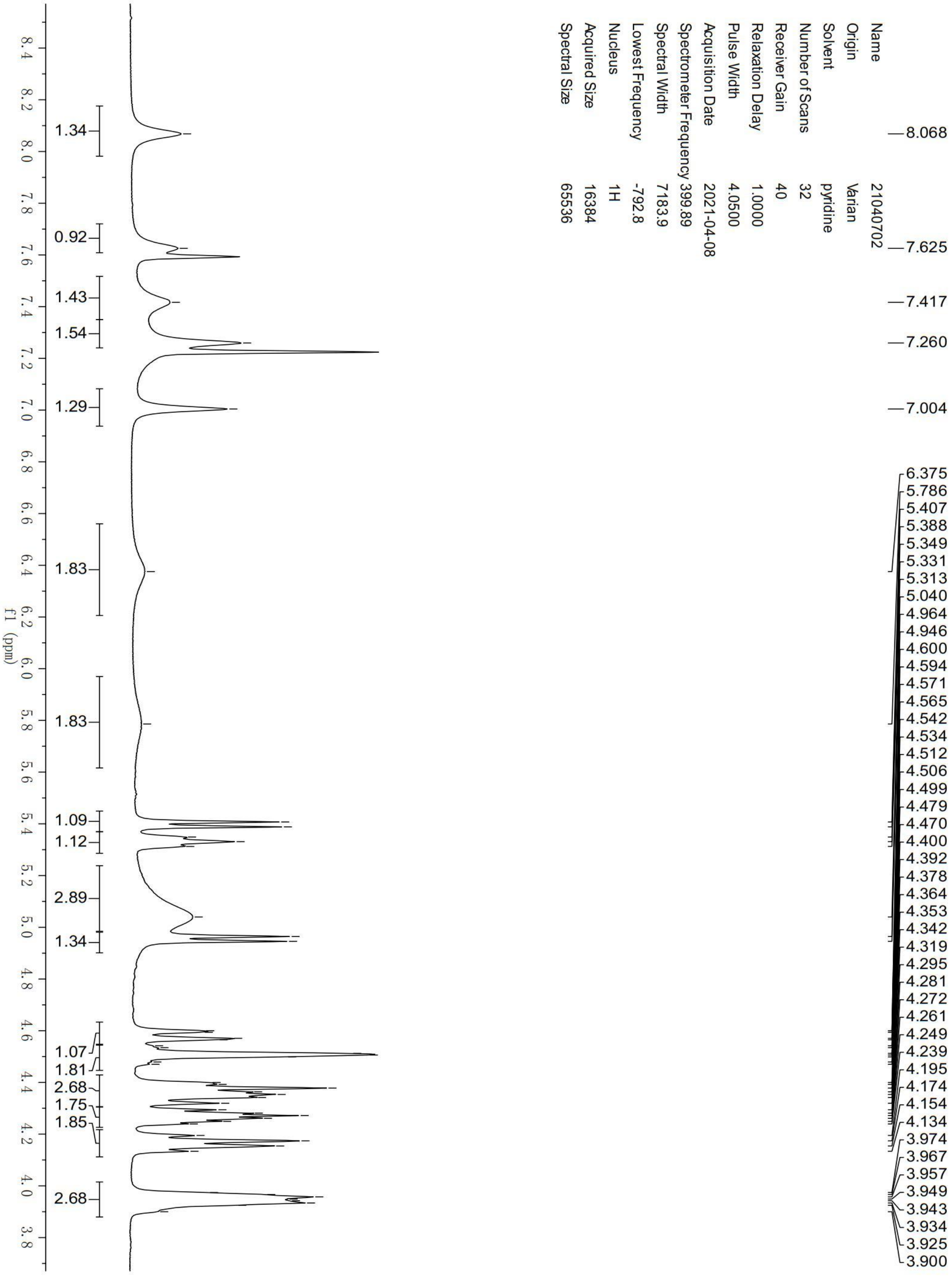
Name 21040702
Origin Varian
Solvent pyridine
Number of Scans 32
Receiver Gain 40
Relaxation Delay 1.0000
Pulse Width 4.0500
Acquisition Date 2021-04-08
Spectrometer Frequency 399.89
Spectral Width 7183.9
Lowest Frequency -792.8
Nucleus 1H
Acquired Size 16384
Spectral Size 65536
8.068
7.625
7.417
7.260
7.004
6.375
5.786
5.407
5.388
5.349
5.331
5.313
5.040
4.964
4.946
4.600
4.594
4.571
4.565
4.542
4.534
4.512
4.506
4.499
4.479
4.470
4.400
4.392
4.378
4.364
4.353
4.342
4.319
4.295
4.281
4.272
4.261
4.249
4.239
4.195
4.174
4.154
4.134
3.974
3.967
3.957
3.949
3.943
3.934
3.925
3.900
1.34
0.92
1.43
1.54
1.29
1.83
1.83
1.09
1.12
2.89
1.34
1.07
1.81
2.68
1.75
1.85
2.68
f1 (ppm)

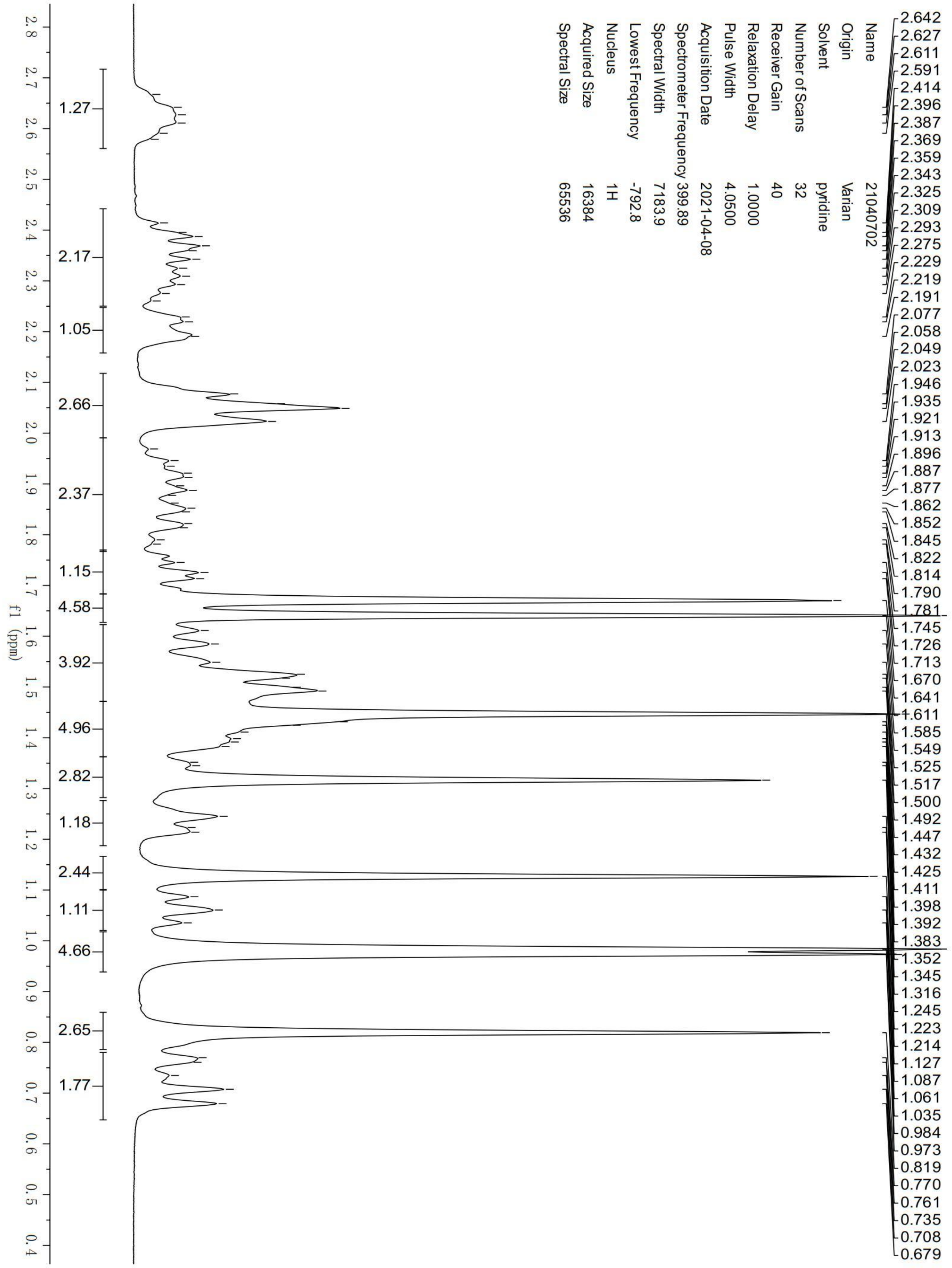
Name 21040702
Origin Varian
Solvent pyridine
Number of Scans 32
Receiver Gain 40
Relaxation Delay 1.0000
Pulse Width 4.0500
Acquisition Date 2021-04-08
Spectrometer Frequency 399.89
Spectral Width 7183.9
Lowest Frequency -792.8
Nucleus 1H
Acquired Size 16384
Spectral Size 65536
2.642 2.627 2.611 2.591 2.414 2.396 2.387 2.369 2.359 2.343 2.325 2.309 2.293 2.275 2.229 2.219 2.191 2.077 2.058 2.049 2.023 1.946 1.935 1.921 1.913 1.896 1.887 1.877 1.862 1.852 1.845 1.822 1.814 1.790 1.781 1.745 1.726 1.713 1.670 1.641 1.611 1.585 1.549 1.525 1.517 1.500 1.492 1.447 1.432 1.425 1.411 1.398 1.392 1.383 1.352 1.345 1.316 1.245 1.223 1.214 1.127 1.087 1.061 1.035 0.984 0.973 0.819 0.770 0.761 0.735 0.708 0.679
1.27 2.17 1.05 2.66 2.37 1.15 4.58 3.92 4.96 2.82 1.18 2.44 1.11 4.66 2.65 1.77
2.8 2.7 2.6 2.5 2.4 2.3 2.2 2.1 2.0 1.9 1.8 1.7 1.6 1.5 1.4 1.3 1.2 1.1 1.0 0.9 0.8 0.7 0.6 0.5 0.4
f1 (ppm)

Description	Ginsenoside Rh1	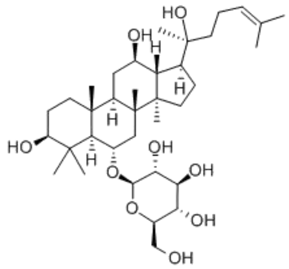
CAS No.	63223-86-9	
MF	C36H62O9	
MW	638.883	

TEST ITEM AND RESULT

Item	Standard	Result	REMARKS
Appearance	powder	Conforms	
Loss on drying	≤ 2.0%	Conforms	
Assay by HPLC	≥ 98%	98.33%	

ATTENTION

Storage	Keep tightly sealed and store under dry and dark conditions. Recommended storage temperature: below 4 。C, special varieties below -20。 C.
Warranty	Two years
Usage	Because some compounds may change at room temperature after dissolved in solvents, please use the dissolved sample early. Chromatographic pure reagents are recommended to dissolve samples for HPLC analysis.
Note	In case of quality problem, please contact us within 15 days after receiving the products.

办公地址：成都市航空港空港国际城一期
生产地址：四川成都天府新区乐至西郊工业园
电话：028-84248411 传真：028-61551164
网址：https://www.herbsubstance.com

色谱条件：照高效液相色谱法测定，以十八烷基硅烷键合硅胶为填充剂；

流 动 相：乙腈-水=32-68,
检测波长:203nm

附：HPLC 图谱

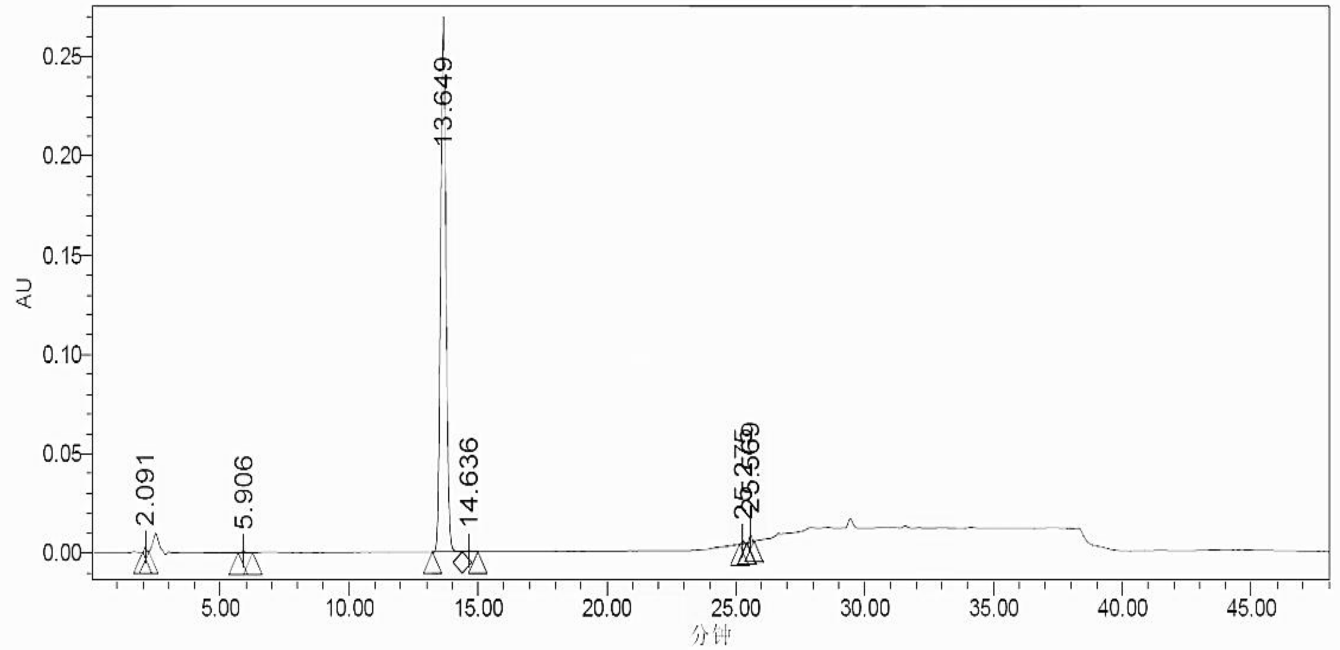

	保留时间(分钟)	面积(微伏*秒)	高度(微伏)	%面积
1	2.091	13793	2332	0.34
2	5.906	9056	803	0.22
3	13.649	4041035	261914	98.33
4	14.636	11340	660	0.28
5	25.275	9655	1503	0.23
6	25.569	24673	3822	0.60

附：核磁

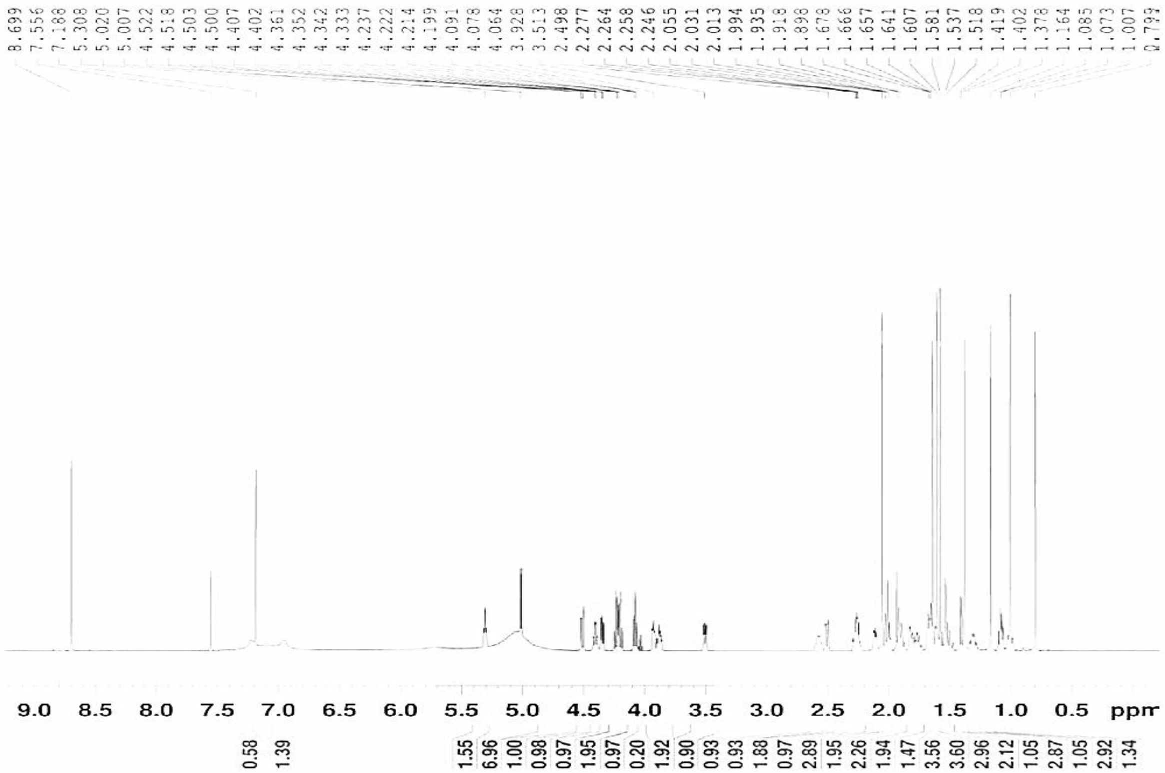
8.699
7.556
7.188
5.308
5.020
5.007
4.522
4.518
4.503
4.500
4.407
4.402
4.361
4.352
4.342
4.333
4.237
4.222
4.214
4.199
4.091
4.078
4.064
3.928
3.513
2.498
2.277
2.264
2.258
2.246
2.055
2.031
2.013
1.994
1.935
1.918
1.898
1.678
1.666
1.657
1.641
1.607
1.581
1.537
1.518
1.419
1.402
1.378
1.164
1.085
1.073
1.007
0.799
9.0 8.5 8.0 7.5 7.0 6.5 6.0 5.5 5.0 4.5 4.0 3.5 3.0 2.5 2.0 1.5 1.0 0.5 ppm
0.58
1.39
1.55
6.96
1.00
0.98
0.97
1.95
0.97
0.20
1.92
0.90
0.93
0.93
1.88
0.97
2.89
1.95
2.26
1.94
1.47
3.56
3.60
2.96
2.12
1.05
2.87
1.05
2.92
1.34

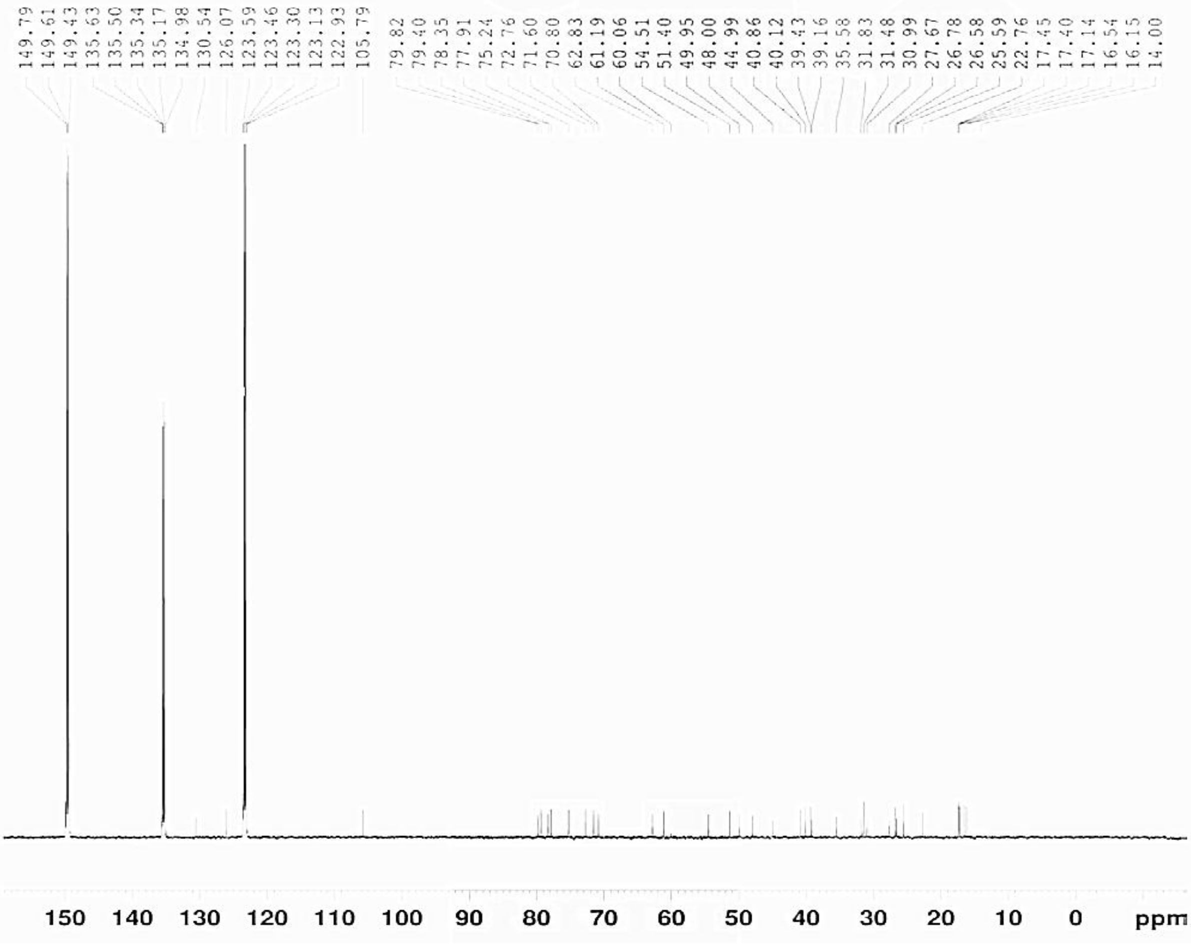
149.79
149.61
149.43
135.63
135.50
135.34
135.17
134.98
130.54
126.07
123.59
123.46
123.30
123.13
122.93
105.79
79.82
79.40
78.35
77.91
75.24
72.76
71.60
70.80
62.83
61.19
60.06
54.51
51.40
49.95
48.00
44.99
40.86
40.12
39.43
39.16
35.58
31.83
31.48
30.99
27.67
26.78
26.58
25.59
22.76
17.45
17.40
17.14
16.54
16.15
14.00
150 140 130 120 110 100 90 80 70 60 50 40 30 20 10 0 ppm

附：质谱

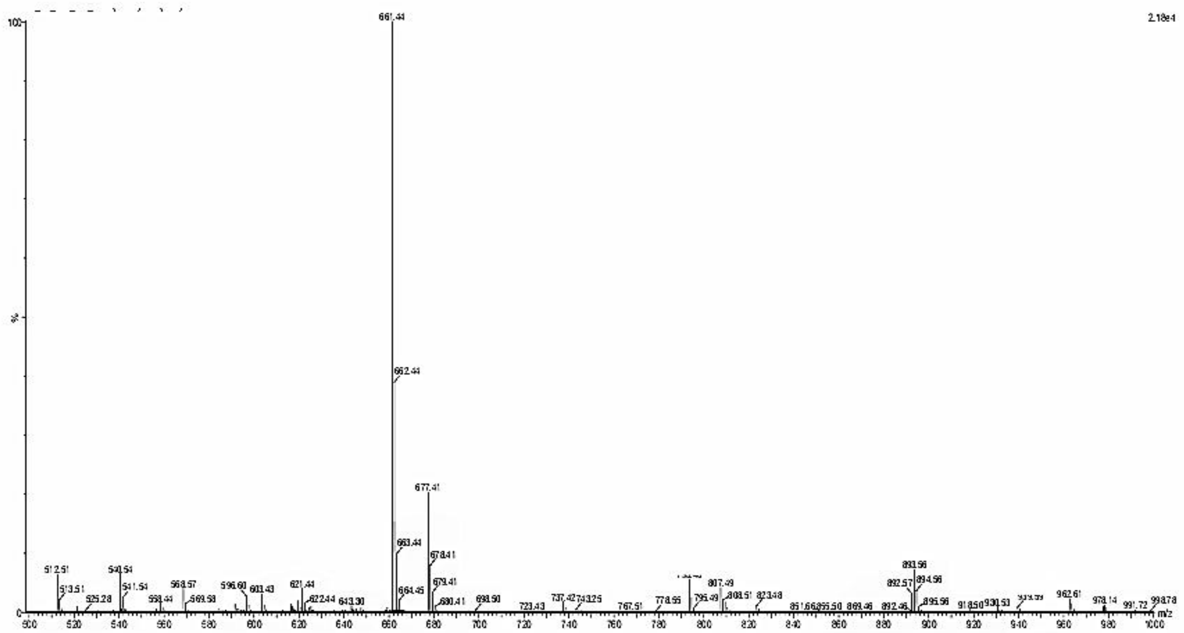

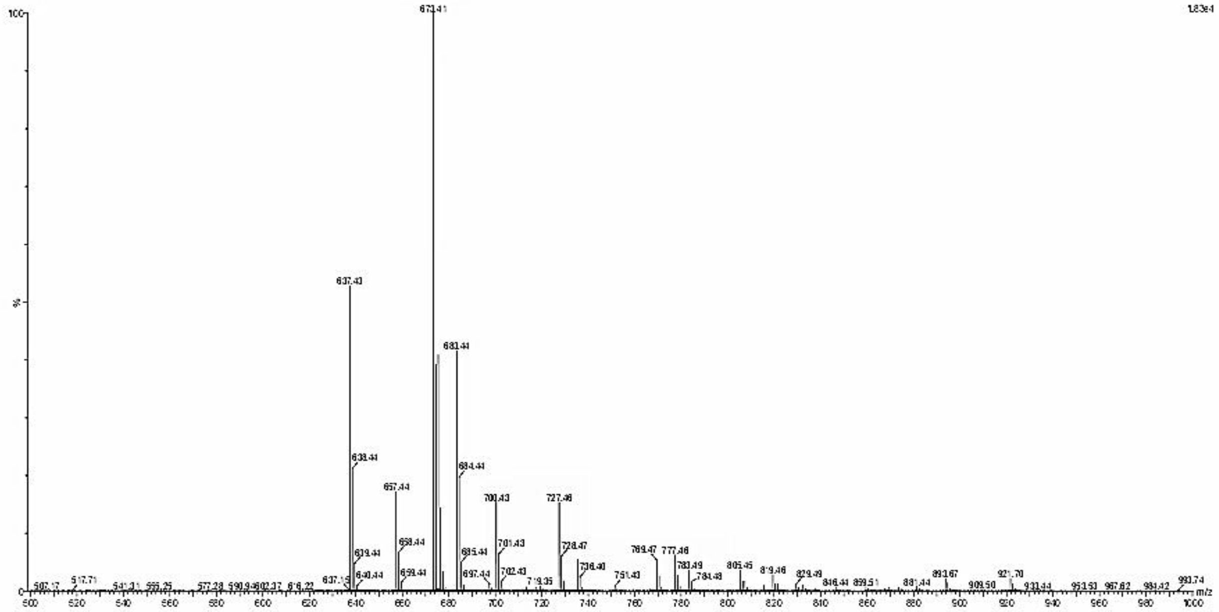

**Description**	Ginsenoside Rh2	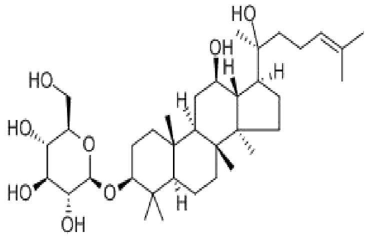
**CAS No.**	78214-33-2	
**MF**	C36H6208	
**MW**	622.87	

TEST ITEM AND RESULT

Item	Standard	Result	REMARKS
**Appearance**	**powder**	**Conforms**	
**Loss on drying**	**≤ 2.0%**	**Conforms**	
**Assay by HPLC**	**≥ 98%**	**99.59%**	

ATTENTION

**Storage**	**Keep tightly sealed and store under dry and dark conditions. Recommended storage temperature: below 4 。C, special varieties below -20。 C.**
**Warranty**	**Two years**
**Usage**	**Because some compounds may change at room temperature after dissolved in solvents, please use the dissolved sample early. Chromatographic pure reagents are recommended to dissolve samples for HPLC analysis.**
**Note**	**In case of quality problem, please contact us within 15 days after receiving the products.**

办公地址：成都市航空港空港国际城一期
生产地址：四川成都天府新区乐至西郊工业园
电话：028-84248411 传真：028-61551164
网址：https://www.herbsubstance.com

色谱条件：照高效液相色谱法测定，以十八烷基硅烷键合硅胶为填充剂；

流 动 相：乙睛-水=55-45 为流动相;
检测波长：203nm，
流　　速：1mL/min

附：HPLC 图谱

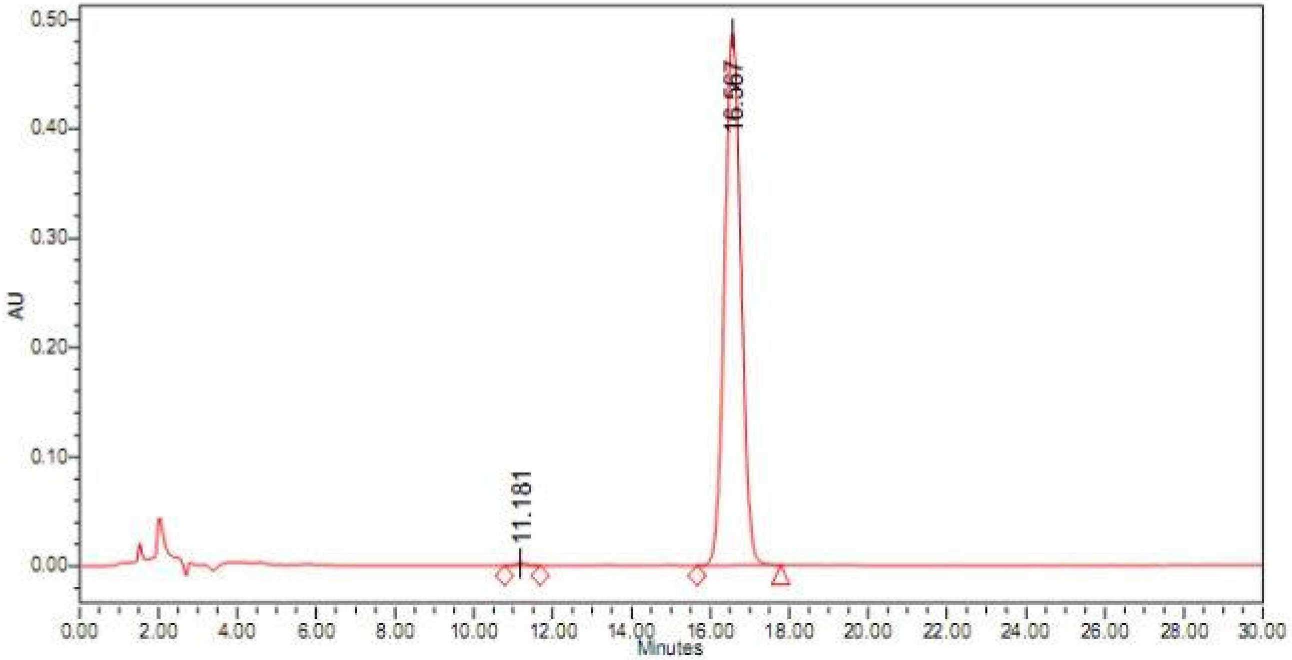

Peak Name:

	Injection	RT	Area	% Area	Height
1	1	16.567	14137955	99.59	486930
2	1	11.181	58500	0.41	2434
Mean		13.874			
Std. Dev.		3.809			
% RSD		27.45			

附：核磁

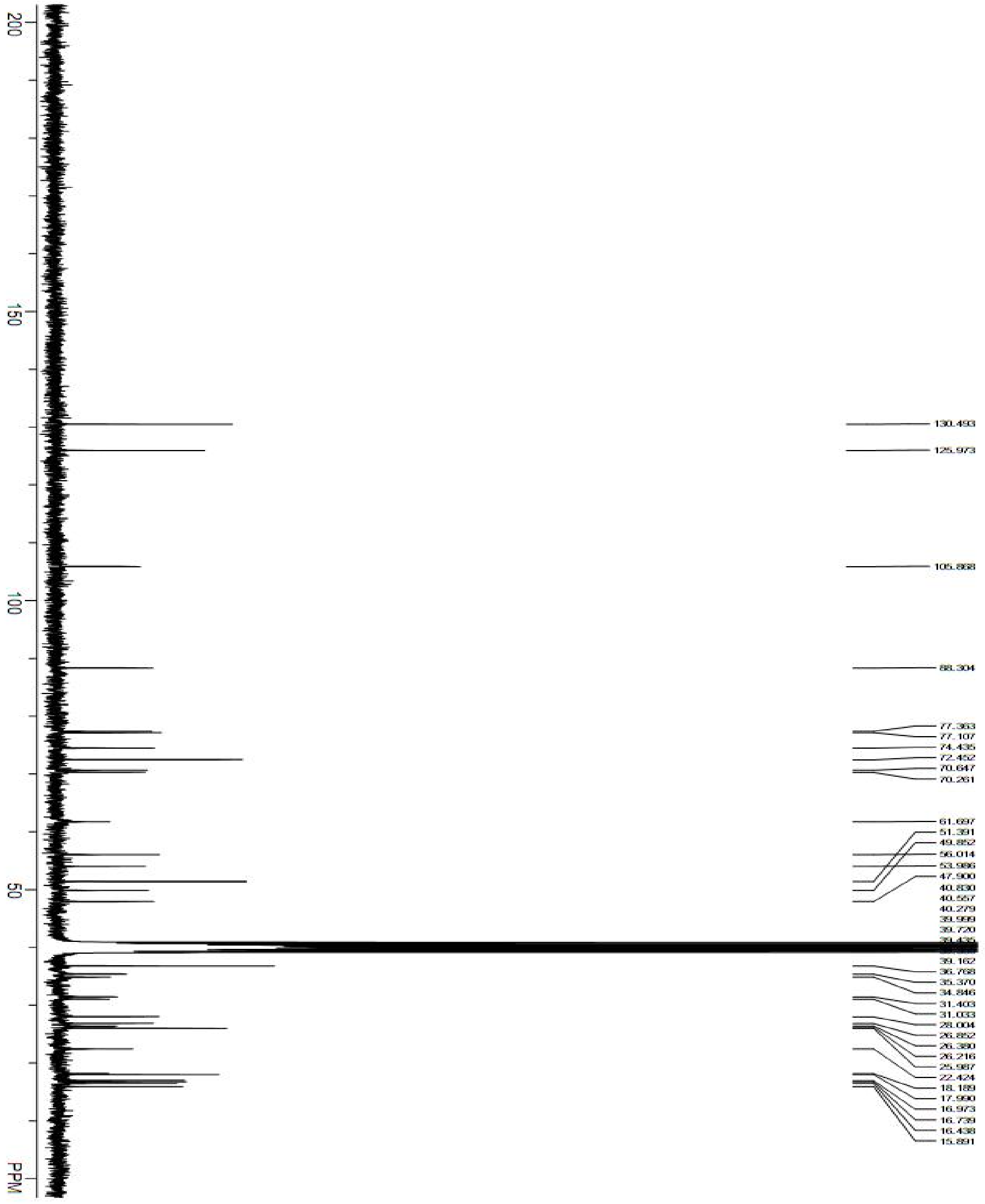
200
150
100
50
PPM
130.493
125.973
105.868
88.304
77.363
77.107
74.435
72.452
70.647
70.261
61.697
51.391
49.852
56.014
53.986
47.900
40.830
40.557
40.279
39.999
39.720
39.162
36.768
35.370
34.846
31.403
31.033
28.004
26.852
26.390
26.216
25.987
22.424
18.189
17.990
16.973
16.739
16.438
15.891

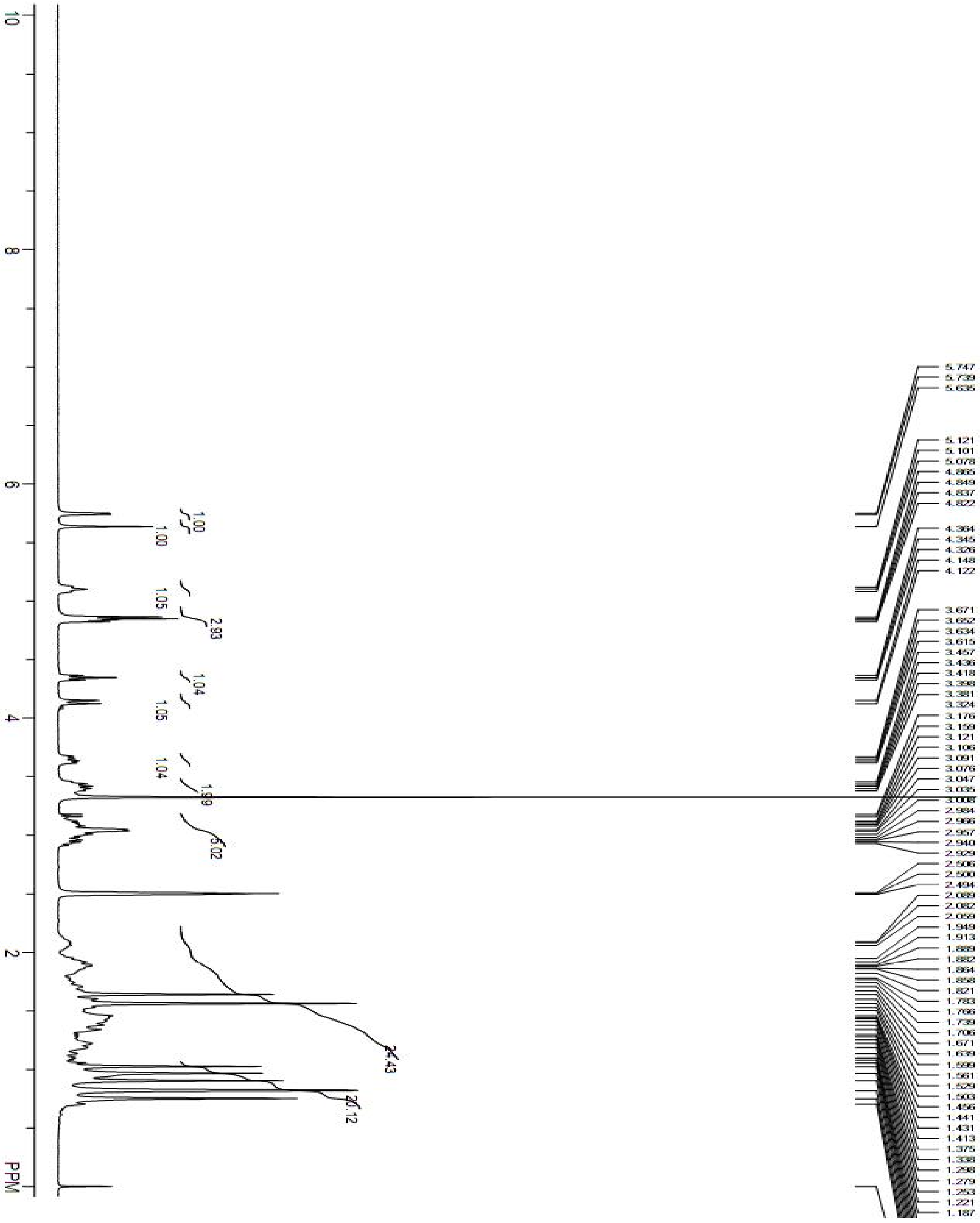
10
8
6
4
2
PPM
1.00
1.00
1.05
2.93
1.04
1.05
1.04
1.99
5.02
24.43
30.12
5.747
5.739
5.635
5.121
5.101
5.078
4.865
4.849
4.837
4.822
4.364
4.345
4.326
4.148
4.122
3.671
3.652
3.634
3.615
3.457
3.436
3.418
3.398
3.381
3.324
3.176
3.159
3.121
3.106
3.091
3.076
3.047
3.035
3.008
2.984
2.966
2.957
2.940
2.929
2.506
2.500
2.494
2.089
2.082
2.059
1.949
1.913
1.889
1.882
1.864
1.858
1.821
1.783
1.766
1.739
1.706
1.671
1.639
1.599
1.561
1.529
1.503
1.456
1.441
1.431
1.413
1.375
1.338
1.298
1.279
1.253
1.221
1.187

Description	Ginsenoside Ro	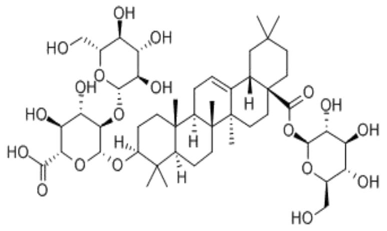
CAS No.	34367-04-9	
MF	C48H76019	
MW	957.109	

TEST ITEM AND RESULT

Item	Standard	Result	REMARKS
Appearance	powder	Conforms	
Loss on drying	≤ 2.0%	Conforms	
Assay by HPLC	≥ 98%	99.2%	

ATTENTION

Storage	Keep tightly sealed and store under dry and dark conditions. Recommended storage temperature: below 4 。C, special varieties below -20。 C.
Warranty	Two years
Usage	Because some compounds may change at room temperature after dissolved in solvents, please use the dissolved sample early. Chromatographic pure reagents are recommended to dissolve samples for HPLC analysis.
Note	In case of quality problem, please contact us within 15 days after receiving the products.

办公地址：成都市航空港空港国际城一期
生产地址：四川成都天府新区乐至西郊工业园
电话：028-84248411 传真：028-61551164
网址：https://www.herbsubstance.com

色谱条件：照高效液相色谱法测定，以十八烷基硅烷键合硅胶为填充剂；

流 动 相：乙腈：0.05%磷酸水 0-15MIN 25:75--50:50 15-30MIN 50:50，
检测波长：203NM

附：HPLC 图谱

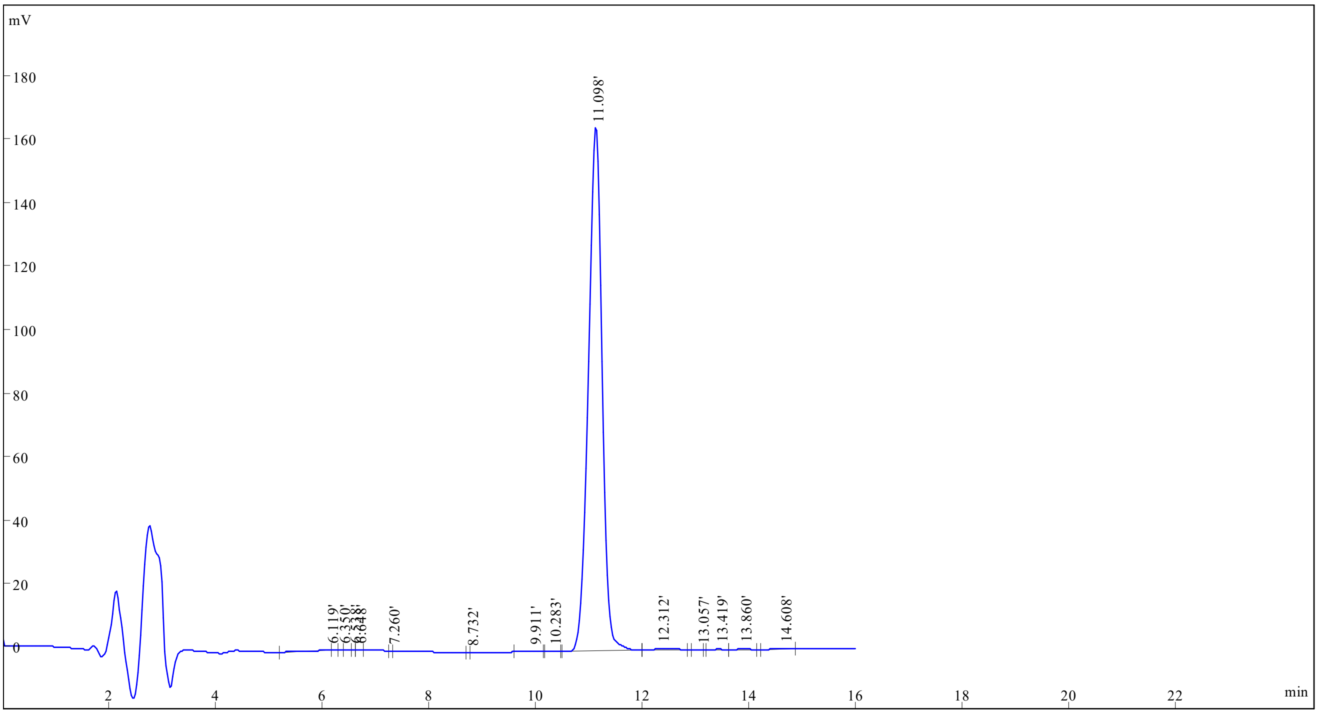

序号	保留时间	名称	浓度	峰面积
1	6.119		0.06621	1978
2	6.350		0.0009003	27
3	6.538		0.0007765	23
4	6.648		0.0009514	28
5	7.260		0.0008986	27
6	8.732		0.0007915	24
7	9.911		0.138	4123
8	10.283		0.008804	263
9	11.098		99.2	2963890
10	12.312		0.4117	12300
11	13.057		0.006284	188
12	13.419		0.04732	1414
13	13.860		0.06461	1930
14	14.608		0.0584	1745
总计			100	2987960

上海源叶生物科技有限公司
Shanghai yuanye Bio-Technology Co., Ltd
电话：400-666-5481 传真：021-55068248
网址：www.shyuanye.com
邮箱：3008007435@qq.com

质量检验报告
CERTIFICATE OF ANALYSIS

产品名称(Product Name)：人参总皂苷

英文名称(English Name）：Total Ginsenoside

储存条件(Storage Condition)：RT

货号(Item No)：S25997 批号 (Lot. Number)：D12IS234679

检测日期(Date of Testing)：2023-12-12 复测日期(Date of Retesting)：2026-12-11

分析项目<br>SPECIFICATION PROPERTIES	技术指标<br>STANDARD	实测结果<br>RESULTS
Appearance	Yellow to brown powder	Brown powder
Assay	80% min	82.56%
Solubility	10mg/mL, MeOH	Clear ,Yellow solution

Quality Assurance：Xiaoyong Tan Quality Control：Yunfei Zhu

上海源叶生物科技有限公司 质检专用章
